# A Graphical Modelling Approach to the Dissection of Highly Correlated Transcription Factor Binding Site Profiles

**DOI:** 10.1371/journal.pcbi.1002725

**Published:** 2012-11-08

**Authors:** Robert Stojnic, Audrey Qiuyan Fu, Boris Adryan

**Affiliations:** 1 Cambridge Systems Biology Centre, University of Cambridge, Cambridge, United Kingdom; 2 Department of Genetics, University of Cambridge, Cambridge, United Kingdom; 3 Department of Physiology, Development and Neuroscience, University of Cambridge, Cambridge, United Kingdom; University of Washington, United States of America

## Abstract

Inferring the combinatorial regulatory code of transcription factors (TFs) from genome-wide TF binding profiles is challenging. A major reason is that TF binding profiles significantly overlap and are therefore highly correlated. Clustered occurrence of multiple TFs at genomic sites may arise from chromatin accessibility and local cooperation between TFs, or binding sites may simply appear clustered if the profiles are generated from diverse cell populations. Overlaps in TF binding profiles may also result from measurements taken at closely related time intervals. It is thus of great interest to distinguish TFs that *directly* regulate gene expression from those that are *indirectly* associated with gene expression. Graphical models, in particular Bayesian networks, provide a powerful mathematical framework to infer different types of dependencies. However, existing methods do not perform well when the features (here: TF binding profiles) are highly correlated, when their association with the biological outcome is weak, and when the sample size is small. Here, we develop a novel computational method, the Neighbourhood Consistent PC (NCPC) algorithms, which deal with these scenarios much more effectively than existing methods do. We further present a novel graphical representation, the Direct Dependence Graph (DDGraph), to better display the complex interactions among variables. NCPC and DDGraph can also be applied to other problems involving highly correlated biological features. Both methods are implemented in the R package *ddgraph*, available as part of Bioconductor (http://bioconductor.org/packages/2.11/bioc/html/ddgraph.html). Applied to real data, our method identified TFs that specify different classes of cis-regulatory modules (CRMs) in Drosophila mesoderm differentiation. Our analysis also found depletion of the early transcription factor Twist binding at the CRMs regulating expression in visceral and somatic muscle cells at later stages, which suggests a CRM-specific repression mechanism that so far has not been characterised for this class of mesodermal CRMs.

## Author Summary

Transcription factors (TFs) are proteins that bind to DNA and regulate gene expression. Recent technological advances make it possible to map TF binding patterns across the whole genome. Multiple single-gene studies showed that combinatorial binding of multiple transcription factors determines the gene transcriptional output. A common naive assumption is that correlated binding profiles may indicate combinatorial binding. However, it has been found that many TFs bind to distinct hotspots whose role is currently unclear. It is thus of great interest to find transcription factor combinations whose correlated binding is causally most immediate to gene expression. Building upon theories of statistical dependence and causality, we develop novel graphical modelbased algorithms that handle highly correlated transcription factor binding profiles more efficiently and reliably than existing algorithms do. These algorithms can also be applied to other biological areas involving highly correlated variables, such as the analysis of high-throughput gene knock-down experiments.

## Introduction

A major area in genome research is understanding how the regulatory information is encoded. Work over the past few decades has resulted in the notion of a combinatorial regulatory code: the concerted binding of a context-specific set of transcription factors (TFs) to regulatory sequences, which is crucial for proper gene expression. Studies of a handful of single genes and their few well-characterised enhancers prevailed in the early days (see [1] for review). The traditionally experimental dissection of enhancers allowed the placing of TFs within a regulatory hierarchy. A canonical example of this traditional dissection is the identification of the various stripe enhancers of the *Drosophila* even-skipped gene that respond to different TFs involved in early patterning (see [2], [3] for review). With the advent of genome-wide detection methods, hundreds of genome-wide TF binding and histone modification profiles have been generated [4]–[6] with the aim of deciphering the combinatorial regulatory code at the global level. Whereas the inference of the regulatory code may greatly benefit from having additional data, such as the expression patterns of the genes of interest under mutant conditions, it is often difficult to collect at the genome level. In the absence of such additional data, a typical strategy is to assume that correlation in TF binding indicates functional interaction between TFs, and to perform correlation-based analyses, such as enrichment analysis (see [7] for a review of strategies in analysing multiple TF binding profiles). However, recent studies provide evidence for so-called “hotspots” to which many interacting or non-interacting TFs may bind [6], [8], [9], which leads to high correlations among binding profiles of both functionally “relevant” and functionally “irrelevant” TFs. It remains a significant challenge to distinguish relevant and important TFs from the others in the understanding of the combinatorial regulatory code.

Similar to the gene regulation problem described above, many other biological problems involve highly-correlated features and high correlation does not necessarily indicate functional relevance. Machine learning approaches, especially classification methods, have been developed to use the measurements of these features (or “explanatory variables”) to predict biological outcomes (or “target variables”), e.g. using core promoter DNA motifs to predict transcription start site locations [10] or using DNA motifs and transcript structures to predict splicing patterns [11]. Although these approaches may produce robust predictions, they do not distinguish which features directly or indirectly influence the biological outcome. Other machine-learning approaches such as standard feature selection methods (see [12] for review) are also not appropriate for this kind of inference in the general case [13], [14].

In contrast, graphical models (GM) [15] encompass a broad class of tools that infer the joint probability distribution of the variables in the network (or graph), and distinguish direct from indirect interactions under broad assumptions. Graphical models achieve this distinction through the notion of conditional independence, which is explained in the Results section. Bayesian networks, also known as Directed Acyclic Graphs (DAGs), are a type of graphical model that further permit the interpretation of causality of the inferred interactions.

Two concepts are particularly important in the theory of Bayesian networks: the causal neighbourhood and the Markov blanket. Specifically, if there is a directed edge from variable A to the target variable T in the network, then variable A is defined as the causal parent of T. If the directed edge goes from T to A, then A is the causal child of T. The causal neighbourhood of the target variable consists of the causal parents and causal children of the target variable. It is thus the set of variables that are most “causally immediate” for the target variable. The Markov blanket of the target variable T contains its causal neighbourhood as well as other causal parents of T's causal children (these other causal parents are T's causal spouses). From the information-theoretical perspective, the Markov blanket contains all the information about the target variable [15], [16].

In terms of statistical inference, existing algorithms for inferring Bayesian networks can be broadly classified into constraint-based, score-based and hybrid algorithms [17]. Constraint-based algorithms perform statistical tests for conditional independence, whereas score-based algorithms estimate the most (or highly) likely joint distribution of the variables in the network. Hybrid algorithms are a combination of the other two, initialising a score-based search with a network inferred by a constraint-based algorithm.

In this paper we develop a novel constraint-based graphical model method, the Neighbourhood Consistent PC (NCPC) algorithms, to infer the causal neighbourhood and the Markov blanket of a target variable. Through synthetic data, we demonstrate that our algorithm has superior performance to existing algorithms when the variables are highly correlated, the data of the target variable is sparse, and the coupling of the target variable and other variables is weak.

We also develop a novel graphical representation, the Direct Dependence Graph (DDGraph), which can represent the dependence patterns inferred from the NCPC algorithms. This representation is broader than the common representation in DAGs, and is useful for exploratory analyses of NCPC results. In particular, the DDGraph shows the conditional independencies in the data even if the underlying network is cyclic or non-faithful to a DAG. Both NCPC and DDGraph are implemented in the R package ddgraph, which is part of Bioconductor (http://bioconductor.org/packages/2.11/bioc/html/ddgraph.html).

Applying our algorithm to genome-wide TF profiles and expression profiles of cis-regulatory modules (CRMs) published in [18] provides novel insight into the transcriptional regulation during mesoderm differentiation in Drosophila embryonic development. We identify not only known TFs that are relevant for specific CRM classes, but also a potentially CRM-specific repression mechanism that has not been suggested before. Although we focus on gene regulation in our paper, our algorithm is applicable to other scenarios discussed earlier that involve highly correlated biological features.

## Results

### Direct and indirect dependencies

We illustrate the concepts of direct and indirect dependencies in terms of the combinatorial binding code of transcription factors. Our aim is to identify transcription factors that *directly* influence the regulatory output of a set of CRMs. Consider the following example. Transcription factor A binds to the CRM of a number of genes and thus directly regulates these target genes, whereas transcription factor B binds to several CRMs where A also binds (perhaps because of chromatin structure), but does not regulate the target genes of A. Therefore, A and B have overlapping binding profiles, and both appear to be associated with gene expression changes. However, the apparent effect of B can be explained away by the effect of A. This means that, if we divide the CRMs into those bound by A and those not bound by A, the binding of B is not associated with gene expression changes in either group. Mathematically speaking, B and the genes are *conditionally independent* given A, suggesting that the effect of B is at most indirect. In contrast, if we divide the CRMs into those bound by B and those not bound by B, the binding of A is still associated with gene expression changes in either or both groups. Mathematically speaking, A and the genes are *dependent given* B, suggesting that the effect of A is direct. Detecting conditional independence is thus central in separating direct from indirect effect [15]. Incidentally, when we consider all the CRMs together, both A and B can be associated with (or equivalently, *marginally dependent* with) the genes.

Below we formally define the types of statistical dependencies our NCPC algorithm and its extension detect. We use *X_i_*, a binary vector, to represent the binding states of the *i*-th TF at a set of CRMs. We use *T*, also a binary vector, to represent the expression states of the genes with which the CRMs are associated. We denote the set of all *m* TF binding profiles as 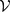, such that 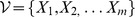. As mentioned in Introduction, *T* is the target variable or outcome, and the *X*s are the explanatory variables or features. Consistent with standard notation, we use symbol 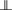 to represent “marginally independent”, and symbol 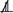 to represent “marginally dependent”. We also use symbol | to represent “conditioning on”. Bold capital letter **S** indicates a subset of 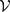, whereas **S**(*X_i_*) indicates a subset of 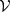 that does not include *X_i_*, i.e., 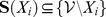.

#### Definition 1

*Variables X_i_ and T are* ***directly*** *dependent if X_i_ and T are marginally dependent (i.e., X_i_* 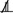 *T)* as well as *dependent when conditioning on any subset* **S**(*X_i_*) *of* 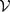 *that does not include X_i_. That is, it holds that X_i_* 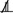 *T* | **S**(*X_i_*).

#### Definition 2

*Variables X_i_ and T are* ***conditionally*** *dependent if X_i_ and T are marginally independent (i.e., X_i_* 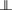 *T), but there exists at least one non-empty subset* **S**(*X_i_*) *such that X_i_* 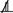 *T* | **S**(*X_i_*).

#### Definition 3

*Variable X_i_ and T are* ***indirectly*** *dependent if X_i_* 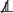 *T, but for at least one non-empty subset* **S**(*X_i_*), *it holds that X_i_* 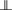 *T* | **S**(*X_i_*).

Note that, in the example above, A and T are directly dependent, whereas B and T are indirectly dependent. When many TFs are involved, often several TFs have similar types of dependence with T. Such collections of TFs are of interest in understanding the complex transcriptional regulatory network and are related to the causal neighbourhood and Markov blanket introduced in the previous section and formally defined below.

#### Definition 4

*A subset* **S** *of* 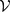 *is a* causal neighbourhood *of T if every variable X_i_ in* **S** *is directly (Definition 1) dependent with T.*

#### Definition 5

*A subset* **S** *of* 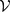 *is a* Markov blanket *of T if every variable X_i_ in* **S** *is either directly (Definition 1) or conditionally (Definition 2) dependent with T.*

As mentioned in the Introduction, whereas the Markov blanket of *T* is the minimal set of explanatory variables that provide all the information about *T*, the causal neighbourhood a subset of the Markov Blanket - contains the main players that have a direct, causal connection with *T*. Note that we aim to identify the causal neighbourhood and do not identify whether the causal neighbourhood is the cause of *T*, or *T* is the cause of the variables in the causal neighbourhood (see Discussion). It means that binding of the TFs in the causal neighbourhood may induce or inhibit certain genes; alternatively, they may be the outcome of the induction or inhibition of certain genes.

### Neighbourhood Consistent PC algorithms

Here we present two versions of the Neighbourhood Consistent PC (NCPC) algorithm, which are based on the PC algorithm [15]. Similar to the PC algorithm (see Supplementary Text), our algorithms perform a series of statistical tests on each explanatory variable to select variables in direct, conditional and indirect dependencies to target *T*. More importantly, our algorithms detect these dependencies even when the explanatory variables *X* are highly correlated among themselves. For example, consider the case where two highly correlated variables *X_i_* and *X_j_* both have direct or conditional dependence with the target variable *T*. However, when testing the null hypothesis of *X_i_* (or *X_j_*) and *T* being independent given *X_j_* (or *X_i_*) for data with a finite sample size, we may not reject this null hypothesis for a given confidence level. Thus, both *X_i_* and *X_j_* may be discarded during the selection procedure. Indeed, the original PC algorithm discards such variables, leading to a low accuracy rate in these scenarios (see Section “Comparison with other algorithms on synthetic data”). To account for potential correlation among variables *X*, our NCPC algorithms specifically check for and retain pairs of variables with the two patterns described below. These patterns depend on the type I error rate *α* of the statistical test used in the algorithm.

#### Candidate pattern 1

*Variables X_i_ and X_j_ have a* ***joint dependency pattern*** *if at level α, (i) they each are* marginally dependent *with T; (ii) X_i_ and T are conditionally independent given X_j_ and* **S**, *and (iii) X_j_ and T are conditionally independent given X_i_ and* **S**, *where* **S** *is any (possibly empty) subset of* 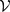, *excluding X_i_ and X_j_. X_i_ and X_j_ in this pattern are candidates for having direct dependency with T.*

#### Candidate pattern 2

*Variables X_i_ and X_j_ have a* ***conditional joint dependency pattern*** *if, at level α, (i) they each have* conditional dependency *with T, (ii) X_i_ and T are conditionally independent given X_j_ and* **S**, *and (iii) X_j_ and T are conditionally independent given X_i_ and* **S**, *where* **S** *is a subset of* 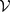 *including the variables X_i_, X_j_ are conditional on, and possibly other variables (excluding X_i_ and X_j_). X_i_ and X_j_ in this pattern are candidates for having conditional dependency with T.*

Although these candidate patterns are mathematically inconsistent (see proof in Supplementary Text), we show in the subsequent section on synthetic data that these patterns can arise in applications with highly correlated variables, and thus should not be discarded.

Between the two versions, the basic NCPC algorithm, shown in Box 1, infers only the causal neighbourhood, retaining variables possibly in direct and indirect dependence with *T*, as well as those in the joint dependency pattern. The NCPC* algorithm, which is the extended version, infers the Markov blanket, retaining in addition variables possibly in conditional dependence and those in the conditional joint dependency pattern. The differences between the two versions will be explained below. See details of the two versions in Supplementary Text.

**Box 1. NCPC Algorithm**

Input:

- Matrix *X* with columns representing different variables (*X*_1_, *X*_2_, …*X_m_*) and rows representing observations.
- Column vector *T* of target variable values, with observations corresponding to those of *X*.
- Conditional independence test appropriate for the dataset

Algorithm:

1. Initialise a set of direct dependence candidates **C** with all *X_i_* marginally dependent with *T*
2. Let *n* = 1
3. Repeat:
  - Enumerate all subsets **S** of size *n* from candidate set **C**
  - For every *X_i_*, if *X_i_* is conditionally independent of *T* given any of the subsets **S**, remove it from the set of candidates
  - Set *n* = *n*+1
  - Break out of the loop if *n* is greater than number of candidates **C**, or, stopping criterion is met
4. Label candidates **C** as having *direct* dependence
5. Systematically check for joint pattern of dependence in tests performed in Step 3
6. If *X_i_* is conditionally independent of *T* only in a joint pattern, label as having *joint* dependence
7. Label all variables removed in Step 3 not having joint dependence as having *indirect* dependence
8. Label all remaining variables as having *no* dependence
9. Return calls for each of the variables in *X*

The NCPC* algorithm differs from the NCPC algorithm in two main ways. Firstly, during the initialisation step, in addition to the candidate set *C* of *X*s marginally dependent with *T*, NCPC* also includes *X*s that are dependent with *T* given variables in *C*. Secondly, NCPC* checks for conditional dependence for individual *X*s as well as conditional joint dependency patterns for pairs of *X*s.

The NCPC and NCPC* algorithms have similar computational complexity to the PC algorithm. That is, in the worst case, the number of required tests increases exponentially with the size of the causal neighbourhood (NCPC) or that of the Markov blanket (NCPC*), although in real life applications, the size of the causal neighbourhood and that of the Markov blanket of *T* are often small. Multiple testing correction [19], [20] can be used as suggested for the PC algorithm [21] (see Supplementary Text for details).

As local network reconstruction algorithms our NCPC algorithms assume that there are no hidden variables or directed cycles (i.e., feedback loops) in the Markov blanket of *T*, although hidden variables or directed cycles may exist in other parts of the system. In Discussion, we examine the impact of deviations from these assumptions.

Assuming an infinite sample size, a perfect statistical test (“conditional independence oracle”) and a dependence structure faithful to a DAG without hidden (i.e. unmeasured) variables, the NCPC* algorithm can correctly label all the variables in the network; that is, this algorithm is asymptotically correct for a distribution faithful to a DAG (see [15] and [22] for similar discussions on asymptotic correctness). This is because all the causal spouses of the target variable *T* enter the candidate list in Steps 1 and 2, such that the set of candidates contains the whole Markov blanket of *T*. Conditional on the whole Markov blanket, all the remaining variables can then be correctly labelled as in indirect dependence. In contrast, the NCPC algorithm is not asymptotically correct, except when there are no variables with conditional dependence, such that the Markov blanket of *T* is identical to its causal neighbourhood. In general the NCPC algorithm may falsely identify indirect dependence as direct dependence. However, as we show in Section “Comparison with other algorithms on synthetic data”, the NCPC algorithm may be empirically more stable than the NCPC* algorithm and thus lead to better results in practice.

### The Direct Dependence Graph

NCPC and NCPC* output labels for the explanatory variables *X*. These labels are the inferred types of dependence, namely “direct”, “indirect” and “joint”, as defined in Definitions 1–3, and the candidate dependency patterns, namely “conditional” and “conditional joint”, as described in Candidate Patterns 1–2. To visualise the inferred dependencies between multiple explanatory variables and the target variable, and especially to represent Candidate Patterns 1–2, we develop a novel graphical representation: the Direct Dependence Graph (DDGraph).

**Figure 1:**
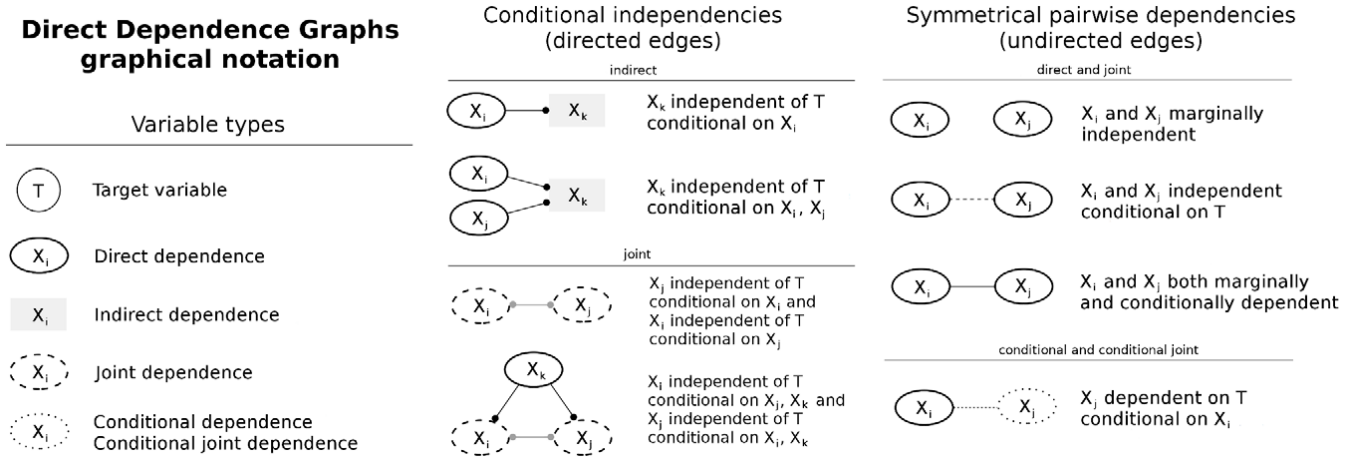
The graphical vocabulary of the DDGraph. The vocabulary consists of five types of nodes and two types of edges. For the edges, directed edges ending with dots indicate conditional independences between *X_k_* and the target variable *T* given *X_i_*. Undirected edges indicate dependencies, which involve *T* in different ways, and for conditional independencies between *X_i_* and *X_j_* given *T*. Consider a case of non-faithful distribution where *T* is an XOR function of *X*1 and *X*2 with carefully set parameters so that from data it looks like *X*1 and *X*2 are marginally independent of *T*. In this case, *X*1 and *X*2 would be conditionally dependent when conditioning on each other. This distribution would be represented as two dotted nodes with a dotted line between them, but disconnected from *T*. This kind of graph signals a non-faithful distribution where the neighbourhood and Markov blanket are not defined by transversing undirected edges from *T*.

DDGraphs use both directed edges (ending in dots) and undirected edges to capture a multitude of dependency patterns with respect to the target variable *T* (see Figure 1 for the graphical vocabulary). For example, directed edge *X_i_* –• *X_j_* represents that *X_j_* is conditionally independent of *T* given *X_i_*. Solid undirected edge *X_i_*–*X_j_* represents that *X_i_* and *X_j_* are both dependent given *T* and marginally dependent. Dashed undirected edge *X_i_* - - *X_j_* represents that *X_i_* and *X_j_* are conditionally independent given *T*. Additionally, black edges indicate dependence patterns that are mathematically consistent, and grey edges indicate the dependence patterns that are inconsistent (e.g. edges in Candidate Patterns 1 and 2).

**Figure 2:**
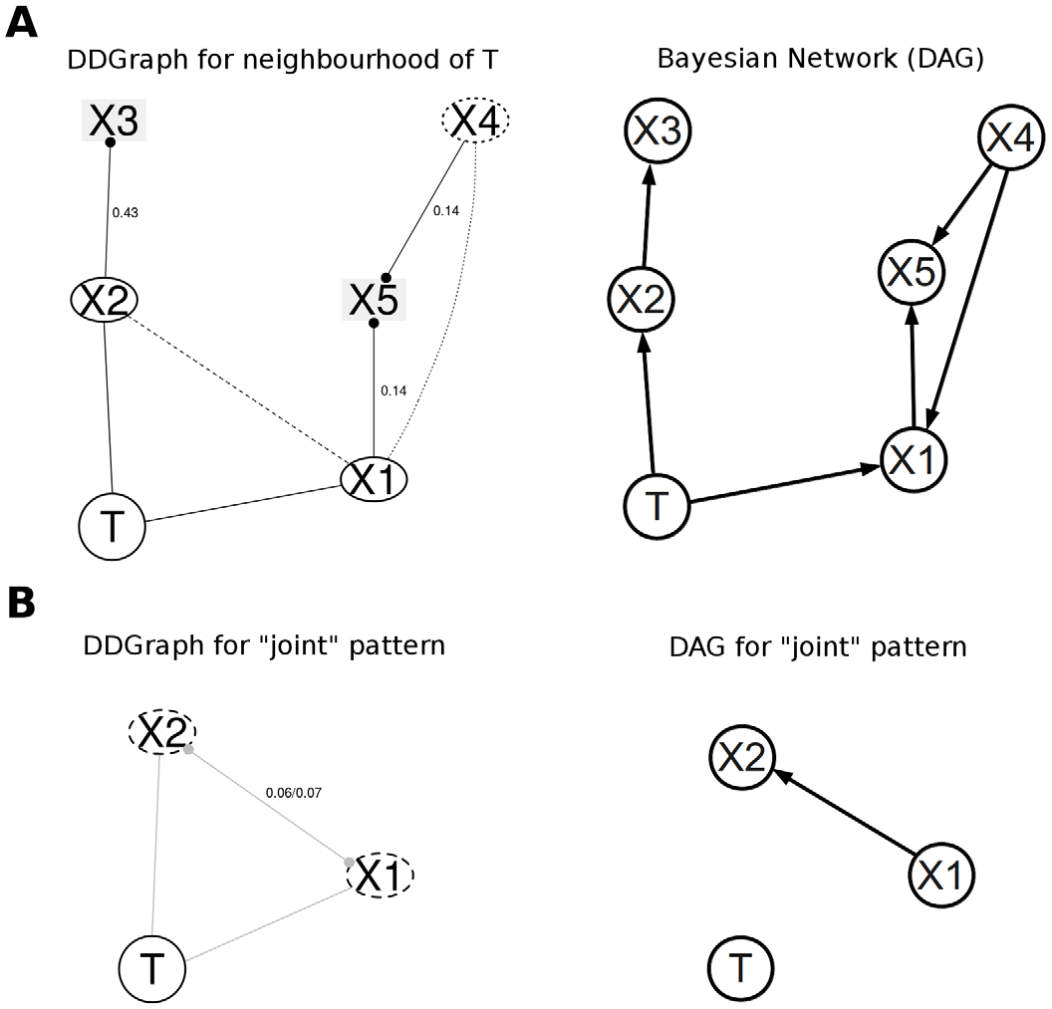
Comparison of DDGraphs and DAGs. (**A**) The causal neighbourhood of the target variable T consists of variables X1 and X2, while T's Markov blanket consists of X1, X2, X4 (in ovals). The remaining variables X3 and X5 have indirect dependence (in rectangles). The DDGraph (left) and the DAG (right) represent the same conditional dependencies. The causal neighbourhood/the Markov blanket and the variable in indirect dependence are distinguishable by the variable shapes in the DDGraph, but have to be inferred in the DAG by following the edges. (**B**) joint dependency patterns representable in the DDGraph (left) cannot be represented by DAGs (right). The DAG shown here represents the conditional independencies between X1 (or X2) and T given X2 (or X1), but it does not represent the marginal dependency between X1 (or X2) and T. Neither this DAG or any other DAG can represent the entire joint dependency pattern.

A DDGraph and a DAG with the same dependence patterns around the target variable *T* is shown in Figure 2A. In a DDGraph, variables connected to the target variable *T* with an undirected edge are in the causal neighbourhood of *T*, and variables reachable from *T* by traversing only undirected edges are in the Markov blanket of *T* (Figure 2A). These variables are also easily recognizable with their oval shapes, whereas variables in indirect dependence with *T* have a rectangular shape. By contrast, the causal neighborhood and Markov blanket in a DAG have to be inferred from the direction of the edges (Figure 2A).

A DDGraph also represents joint and conditional joint dependency patterns, which are mathematically inconsistent and thus impossible to represent with DAGs (Figure 2B). Indeed, DAGs, as well as other factorization-based graphs, such as factor graphs [23], that represent a factorization of a joint probability distribution cannot represent these inconsistent dependency patterns.

### Comparison with other algorithms on synthetic data

We generated synthetic data based on the 15 correlated TF binding profiles in [18]. See Materials and Methods for details on data generation. The target variable *T*, which is a binary vector that contains the expression states of a set of CRMs, is sparse: similar to the real data, only around 10% of CRMs show class-specific expression. We generated data for three sample sizes: 300, 500 and 1000; the sample size in the data of [18] is 310. In addition, we simulated a causal neighborhood of two variables (*X*_1_, *X*_2_) for *T*, and these causal neighbors are weakly correlated with *T* (correlation 0.17–0.25). We simulated data with four levels of correlation between the two causal neighbors: no correlation (0), weak correlation (0.25), strong correlation (0.50; similar to the average correlation of 0.46 we found in the data from [18]), and very strong correlation (0.75).

**Figure 3:**
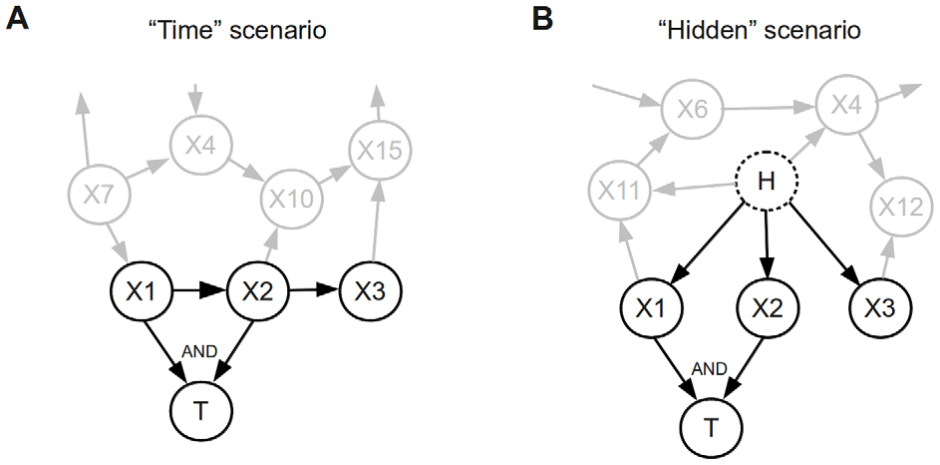
Two scenarios for generating the synthetic data with correlated variables. While the synthetic data were generated for a network of 15 explanatory variables, only variables X1 and X2 have direct dependence with the target variable T, and therefore constitute the causal neighborhood of *T*. Variable X3 is included as the confounding variable. (**A**) The “Time” scenario in which X1, X2 and X3 correspond to three time points with stronger correlation between X1 and X2 and between X2 and X3 than between X1 and X3. (**B**) The “Hidden” scenario in which X1, X2 and X3 are correlated due to a common cause *H* in the network. This common cause is used in data generation, but is not available to algorithms.

We further introduced a third variable (*X*_3_) as the confounding variable in the network and generated correlated data for two realistic scenarios:

- Time - The two causal neighbours (*X*_1_, *X*_2_) and the third variable (*X*_3_) represent the binding profiles of the same TF at three times, such that *X*_1_→*X*_2_→*X*_3_, in which the correlation between *X*_1_ and *X*_3_ is smaller than that between *X*_1_ and *X*_2_ and between *X*_2_ and *X*_3_ (Figure 3A).
- Hidden - The three variables are correlated with a common unobserved cause, e.g., the chromatin and/or cell population structure (represented by *H* in Figure 3B). We set the correlation between any pairs of these three variables to be the same.

With these synthetic data, we focus on the performance of separating direct from indirect dependence and detecting the causal neighbourhood. We applied our NCPC and NCPC* algorithms, at an *α* level of 0.05, to these data. Of the constraint-based algorithms, multiple testing correction has been mathematically and empirically demonstrated only for the PC algorithm [21], [24], [25], therefore, for fair comparison we applied all algorithms, including NCPC/NCPC*, without any multiple testing correction. To investigate the effectiveness of identifying pairs of variables in Candidate Patterns 1 and 2 (see Section “Neighbourhood Consistent PC algorithms”), we applied the NCPC algorithm in two ways: detecting variables only in direct dependence with the target variable, and in addition detecting pairs of variables in joint dependence (Candidate Pattern 1). Similarly, we applied the NCPC* algorithm in two ways: detecting variables only in direct and conditional dependence with the target variable as well as pairs of variables in joint dependence, and detecting, in addition, pairs of variables in conditional joint dependence (Candidate Pattern 2). For comparison, we also applied the following algorithms to the synthetic data: the original PC algorithm [15]; score-based algorithms that infer the whole network, such as Hill-climbing with BIC penalization [26] or with a Dirichlet prior (BDe penalization [27]); other constraint-based algorithms that infer the local structure, such as IAMB [28], FastIAMB [29], InterIAMB [29] and MMPC [30]; as well as a hybrid algorithm MMHC [30].

**Figure 4:**
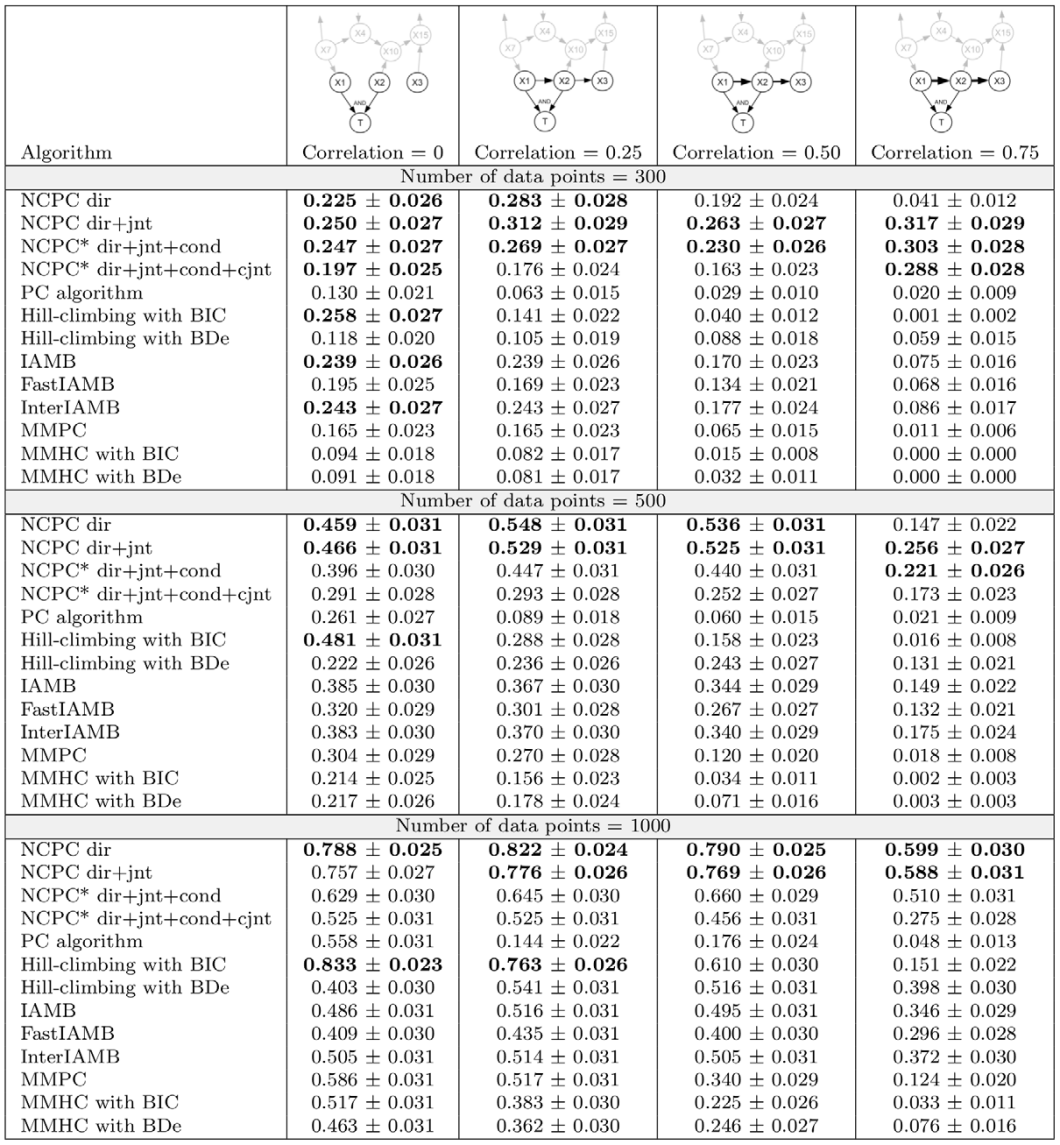
Proportion of correct predictions for the “Time” scenario. Each cell shows the mean proportion of correct predictions (with 95% confidence intervals) averaged over 1000 data sets generated in each case. Highest prediction proportions accounting for variation in the data (pairwise T-tests with a cut-off of 0.001 for the P values) are shown in bold. See Materials and Methods for the generation of the synthetic data and for the calculation of the correct prediction proportion.

We measured the proportion of correct predictions from these algorithms over 1000 data sets generated for each combination of the sample size and correlation in either of the two scenarios. A prediction is correct when only the two causal neighbors and no other variables are identified. These prediction rates for the “Time” scenario are summarized in Figure 4. The prediction rates for the “Hidden” scenario are similar and are summarized in Supplementary Figure S1 in Text S1.

Identifying variables in direct dependence and in joint dependence, the NCPC algorithm (“NCPC dir+jnt”), has the highest (accounting for variation in simulated data) rate of correct predictions amongst all the algorithms in all the cases examined here, except in the biggest dataset with 0 correlation. This superior performance is particularly notable when the correlation between the variables is high and the dataset is small. By including the variable pairs in joint dependence, “NCPC dir+jnt” achieves better performance “NCPC dir” because this inclusion drastically improves recall (corresponding to low false negative rates), especially when the sample size is not large, although the inclusion lowers precision (corresponding to high false positive rates) slightly (see rates of precision and recall defined in Materials and Methods and computed in Supplementary Figures S2 and S3 in Text S1). The comparison of the two implementations of the NCPC algorithm provides some empirical evidence for including pairs of variables at least in Candidate Pattern 1 as candidates for direct dependence. With the sample size as large as 1000, the data are informative enough for “NCPC dir” to perform similarly or even slightly better than “NCPC dir+jnt”. The performance of the two implementations of the NCPC* algorithm, however, is worse than the NCPC algorithm in most cases. This is likely because in order to identify the Markov blanket, which is larger than the causal neighbourhood, the NCPC* algorithm sacrifices the false positive rates more to gain even lower false negative rates. At different levels of correlation, the NCPC and NCPC* algorithms both have more stable precision and recall rates than other algorithms (Supplementary Figures S2 and S3 in Text S1). This may explain why the NCPC and NCPC* algorithms (four implementations) perform better than all the other algorithms.

Increasing the sample size improves the prediction for most algorithms, as we expected. However, when the correlation in the data is 0.75, the NCPC and NCPC* algorithms have lower rates of correct predictions for data with a sample size of 500 than for data with a sample size of 300. This may be due to the *α* level chosen for the statistical test before running the algorithm, especially when the P-values obtained by the NCPC and NCPC* algorithms are close to the value of *α*. A more stringent *α* level such as 0.01 leads to improved performance (Supplementary Figures S4 and S5 in Text S1). This highlights the importance of choosing an appropriate *α* value, and suggests re-running the algorithm with a different *α* level if the P-values obtained are close to the initial *α* value. We recommend the user to inspect the P-values of key conditional independence tests that give rise to the DDGraph and to change the *α* value accordingly.

### Application of NCPC and NCPC* to fly mesoderm development

**Figure 5:**
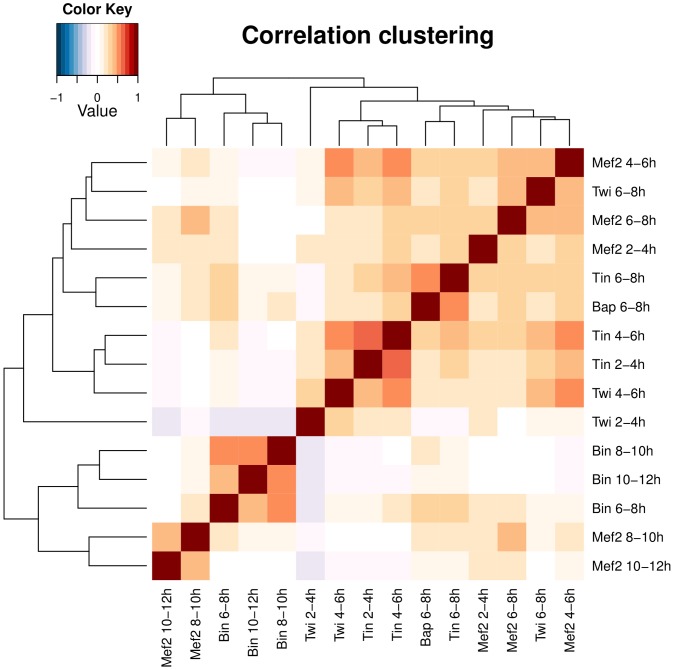
Clustered pairwise correlation matrix of the 15 transcription factor binding profiles over all 310 CRMs. Note that the cluster that consists of Mef2 8–12 h and Bin 6–12 h (lower left corner of the matrix) is anti-correlated with early Twi 2–4 h binding.

Zinzen et al. [18] published an in vivo ChIP-chip temporal binding profiles of key transcription factors that are involved in mesoderm development in fly embryos, as well as the CRM Activity Database (CAD), the largest such database thus far, which contains tissue-specific temporal expression patterns driven by these CRMs. The five key TFs, Twist (Twi), Myocyte enhancer factor 2 (Mef2), Tinman (Tin), Bagpipe (Bap), and Biniou (Bin), were each measured in some or all of five developmental stages, producing 15 correlated TF binding profiles (Figure 5). Zinzen et al. further focused on 310 CRMs from the CAD that have both TF binding and expression data, and classified these CRMs into five classes based on their tissue-specific expression patterns: mesodermal (Meso), mesodermal and somatic muscle (Meso&SM), visceral muscle (VM), visceral and somatic muscle (VM&SM) and somatic muscle (SM).

**Figure 6:**
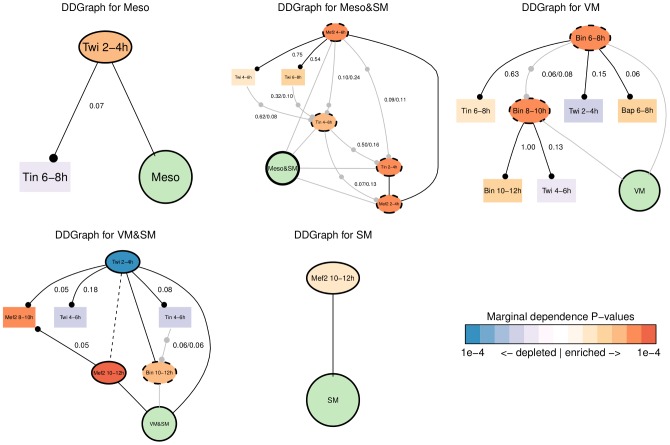
DDGraphs for the 5 CRM classes inferred by the NCPC algorithm at *α* = 0.05. Variables in green circles are target variables. Variables in ovals are inferred causal neighbours. Variables in rectangles are inferred to have indirect dependence with the target. Values on the edges are (unadjusted) P-values from conditional independence tests. The same NCPC algorithm with no multiple testing correction was used as in the synthetic data benchmark. See Figure 1 for the graphical vocabulary.

Here we applied the NCPC and NCPC* algorithms to the same 310 CRMs with the 15 TF binding profiles. The advantage of this dataset is that any computational predictions can be benchmarked against a wealth of previously established biological results. At an *α* level of 0.05, we identified expression class-specific causal neighbourhoods using NCPC (Figure 6 and Supplementary Figure S6 in Text S1). The Markov blankets identified by applying NCPC* (Supplementary Figure S7 in Text S1) are similar to their corresponding causal neighbourhoods. We discusss the biological implications of our inference in the next section.

We also applied other algorithms benchmarked in the previous section to this data set. Hill-climbing with BIC identified a smaller but overlapping set of variables (Supplementary Figure S8 in Text S1), consistent with our results on synthetic data that this algorithm has higher precision but a lower recall rate than our NCPC algorithms. Hillclimbing with BDe identified a bigger but overlapping set of variables (Supplementary Figure S9 in Text S1), also consistent with our results on synthetic data that this algorithm has lower precision than but a similar recall rate to our NCPC algorithms. The IAMB family of methods found either a smaller but overlapping set of variables, or no variables (Supplementary Figures S10, S11 and S12 in Text S1). The original PC algorithm performed similarly to the IAMB methods (Supplementary Figure S13 in Text S1). MMHC produced similar results to those from the ordinary hill-climbing method (Supplementary Figures S14 and S15 in Text S1).

### TF combinatorial code of fly mesoderm development

We applied our method on the dataset of early mesoderm development in the Drosophila embryo [18]. The five transcription factors Twi, Tin, Mef2, Bin and Bap have been previously implicated in mesoderm development of the fly. Among the five TFs we analysed here, Twi, together with another TF Snail, is the earliest marker of mesoderm and is required for mesoderm formation [31]. Tin, a direct target of Twi, is crucial for the differentiation of heart, somatic and visceral mesoderm and is present also in dorsal somatic muscle precursor cells [32]. Mef2, crucial for early muscle differentiation, is present in both visceral and somatic muscle [33]–[35]. Activated by Tin, Bap specifies cells that become the visceral muscle [36], [37]. Finally, Bin is expressed only in visceral muscle cells and is crucial for their differentiation [38].

**Figure 7:**
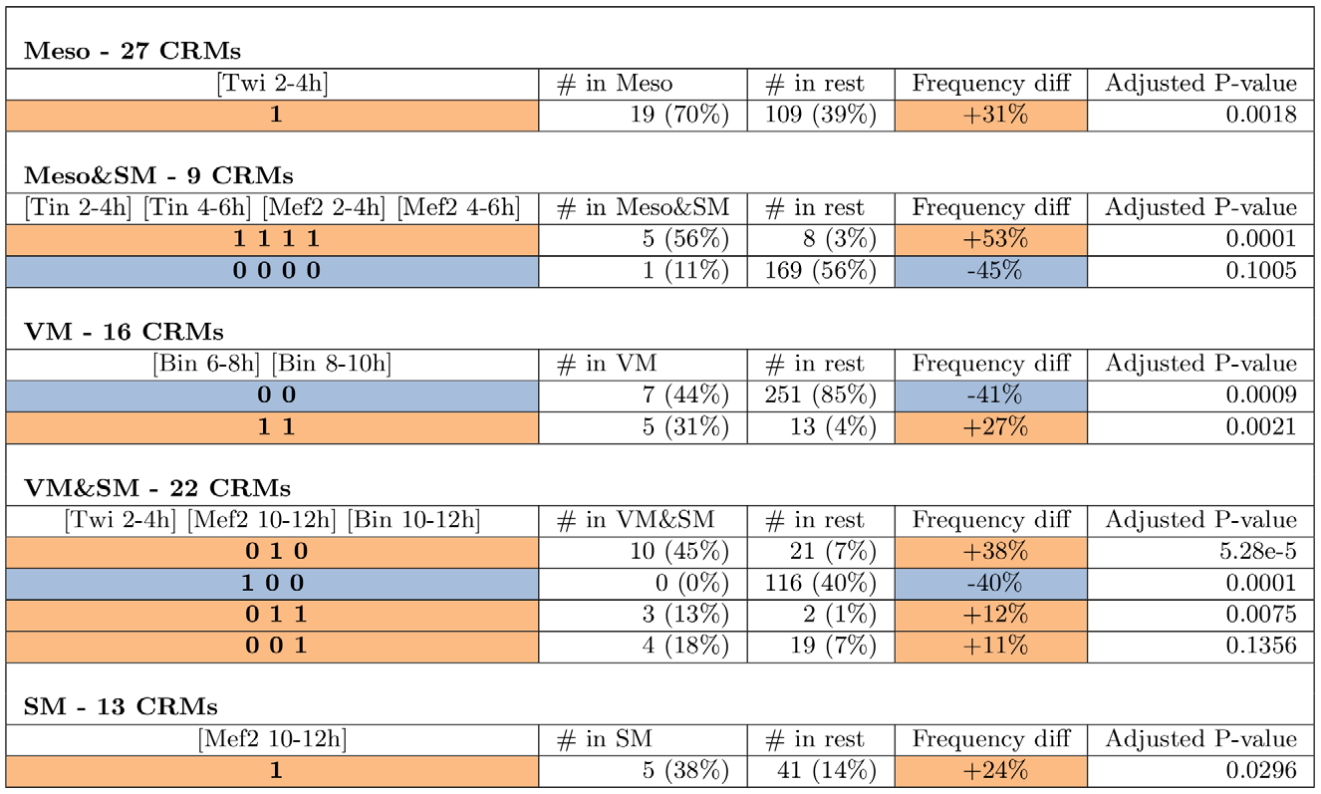
Combinatorial patterns of TFs in inferred causal neighbourhoods. For each combinatorial pattern we show the number of CRMs with this pattern in the CRM class and that in the rest of CRMs (percentages are given in parenthesis). The difference in the two frequencies (CRM class vs rest) and the corresponding P-value are given in the last two columns. P-values were computed from Fisher's exact test for each combination and adjusted for multiple testing using the Benjamini-Hochberg method. See Materials and Methods for details. Frequency differences are colour-coded: blue for decrease in the CRM class, and orange for increase in the CRM class.

After identifying the causal neighbourhood, we further examined which specific TF combinations are enriched or depleted in each of the five expression classes, compared with the rest of the 310 CRMs analysed here (Figure 7). Most of these TF combinations have been established in single-gene studies:

- **Meso** - these CRMs are active only in the early mesoderm (2–6 h). For these we find that Twi 2–4 h binding alone activates these CRMs (Figure 6). This result is not surprising, as Twi is the key regulator of mesoderm development. Note also that Twi 2–4 h is negatively correlated with binding profiles from later stages (Figure 5), perhaps due to changes in the chromatin structure during development, such that the set of early CRMs bound by Twi at 2–4 h are not accessible at later stages.
- **Meso&SM** - these CRMs are active in both early mesoderm (2–6 h) and somatic muscle precursor cells (after 6 h). This CRM class contains only 9 active CRMs, the fewest among the five classes. Tin at 2–4 h and 4–6 h and Mef2 from the same time intervals all have joint dependence with the CRM class activity (Figure 6). Furthermore, the presence of both TFs at both stages are significantly enriched in this CRM class (Figure 7), suggesting that this class of CRMs have a different TF combinatorial code from that for the CRMs active only in early mesoderm (“Meso”). However, Mef2 is not significantly bound after 6 h, and no data is available for Tin after 6 h. Thus, it is unclear which TFs contribute to the somatic activity later on.
- **SM** - these CRMs are active in somatic muscles after 6 h or later in development. This CRM class was difficult to predict with a Support Vector Machine, the approach [18] used. Here we found only Mef2 at 10–12 h to be directly associated with the CRM class activity (Figure 6), although Mef2 at 6–8 h has a P-value just above the *α* level of 0.05 and could have been inferred to pair up with Mef2 at 10–12 h to form the joint dependence pattern. It is likely that for this class of CRMs we are missing some of the key TFs.
- **VM** - these CRMs are active in visceral muscle after 6 h of development. TFs Bin and Bap are known to express only in visceral muscle and are crucial for its development. Thus we would expect both of them to constitute the combinatorial code. We found that Bin at 6–8 h and at 8–10 h are in joint dependence with this CRM class (Figure 6), and that Bin binding at both stages is indeed significantly enriched (Figure 7). These observations together indicate that persistent binding of Bin alone activates this CRM class. Note we did not recover Bap as part of the combinatorial code. By examining the DDGraph we note that Bap is found to have indirect dependence with a P-value just above the threshold (0.06). Furthermore, to our knowledge, the only CRM where Bap binding has been directly proven is the betaTub60D enhancers [39], however the CRM containing this binding site (CRM ID 1443) was annotated with VM&SM activity. Thus, annotation bias might explain why Bap is missing from the combinatorial code at *α* = 0.05.

In addition to previously established regulatory principles outlined above, the genome-wide statistics also suggest a thus far uncharacterized mechanism of prevention of early Twi binding at 2–4 h of embryogenesis for the class of CRMs active in visceral and somatic muscle (VM&SM) at 8–12 h of development. This suggests that these CRMs are selectively shut off during early embryogensis, but are bound later on by tissue-specific transcription factors:

- **VM&SM** - these CRMs are active in visceral and somatic muscle after 8 h of development. It is known that at this stage Mef2 is expressed in both visceral and somatic muscles, while Bin is expressed only in visceral muscles [33]–[35], [38]. We identified that Bin and Mef2 binding at 10–12 h are in joint and direct dependence, respectively, with this CRM class (Figure 6). This is consistent with the important role previously established for these TFs in visceral and somatic muscle development [33]–[35], [38]. Earlier Mef2 binding at 8–10 h has been found to be indirect, but with a P-value barely above the 0.05 threshold (P-value of 0.051). Thus, it is possible that Mef2 binding at 8–10 h is also part of the combinatorial code. In addition, Mef2 binding at 10–12 h alone is significantly associated with activity of this CRM class (Figure 7), we find that although the pattern when both Mef2 and Bin are co-bound is most highly enriched, only a minority of CRMs have this pattern. Instead, most either have only Mef2 binding, or only Bin binding. In fact, the binding of Mef2 and Bin at 10–12 h seems largely independent (Figure 6), suggesting that these TFs may not interact much with each other during visceral and somatic muscle development. We also identified, rather surprisingly, that Twi 2–4 h is in direct dependence with this CRM class, which may suggest a repression mechanism that has not been characterised yet. See further discussion below.

Twi 2–4 h is identified to also have direct dependence with this VM&SM CRM class (Figure 6), and, interestingly, it is the lack of Twi binding at 2–4 h that is significantly associated with the activity of this CRM class. Note that this observation is consistent with the negative correlations between the binding profiles (over all 310 CRMs) of Twi 2–4 h and both Bin and Mef2 at later stages (Figure 5). However, it is unclear how depletion of Twi at an earlier stage leads to activity of these CRMs several hours later. One plausible biological explanation is that these CRMs may be silenced during early embryogenesis (for example, the chromatin they are located in is inaccessible during this stage), and be bound by tissue-specific TFs, such as Bin and Mef2, later. Activation of the CRMs in this class may require concerted efforts, which may be specific to this CRM class, and which may involve remodelling of chromatin or inhibition of early Twi binding. It is also unclear whether additional transcription factors or chromatin remodelling factors are involved in the activation of this CRM class.

## Discussion

In this paper we present a novel graphical model-based method that distinguishes direct from indirect dependencies between explanatory variables (or features) and the target variable. Our NCPC and NCPC* algorithms work particularly well in cases of highly correlated features and of sparse or weak signals, as seen in comparison with other algorithms on synthetic data.

We applied our algorithms to data published in [18], which consist of the 15 transcription-factor binding profiles over 310 CRMs in Drosophila during mesoderm development. Our analysis identified known combinations of TFs associated with expression of different CRM classes. Our analysis also suggests an uncharacterized repression mechanism: depletion of Twist binding at 2–4 h plus presence of tissue-specific factors Mef2 and Bin indicates activity of the CRMs in the visceral and somatic muscle development, through CRM silencing in early embryogenesis and/or chromatin remodelling. Additional TFs may be involved in mesodermal development, and our algorithms can be easily applied to newly available data [40] to improve the local network structures we identified here.

Our NCPC algorithms assume no hidden variables in the Markov blanket of the target variable. This assumption is frequently not met in reality; for example, in the case of the transcriptional regulation, a number of relevant TFs might not have been measured. In that case, a seemingly irrelevant TF might be inferred as a causal neighbour if it is correlated with the unmeasured relevant TF (e.g. due to open chromatin structure). Such a TF would be a “proxy” for the binding of the relevant TF.

Our NCPC algorithms also assume no feedback loops in the Markov blanket of the target variable. This may not be the case in a real biological system. However, if time course data are available and informative enough such that the underlying Markov blanket is acyclic at each time point, then our NCPC algorithms can still be applied (similar to the way we re-analysed the fly mesoderm development data) to identify causal neighbours. Transcriptional responses are typically slow (on the order of minutes [41]) which allows for the data to be collected as time series so that the next time point is a product of the previous time point and thus the dynamics made acyclic in time.

The statistical tests our algorithms perform for the variables in these systems tend to be highly dependent. It is still a challenge to control the false discovery rate for highly dependent tests. We implemented the multiple testing procedure of [21] for controlling the false discovery rate (see Supplementary Text for detail). However, we found that this procedure can be overly conservative and can lead to loss of statistical power, for example even at 0.3 FDR the somatic muscle (SM) class has no causal neighbours (data not shown) although *in-vivo* validation found a weak but predictive signal [18]. Further development in controlling the FDR for dependent tests in network inference is needed.

The NCPC algorithms infer the causal neighbourhood and do not optimise the prediction accuracy of the target variable. Hence, we do not expect these algorithms to be an optimal feature selection procedure for classification. Nonetheless, the NCPC algorithms may in principle be used for feature selection to improve prediction accuracy, for example, by using cross-validation to choose a P-value threshold that minimises the cross-validation error. Directly incorporating the dependence structure in a classifier is still challenging, since it is difficult to robustly estimate higher-order conditional probabilities from small datasets (a Naive Bayesian Classifier has been used in practice; see [42]).

A wealth of genome-wide data have been and are currently produced, featuring binding sites of transcription factors, chromatin marks and RNA levels [6], [9], [43]. Our NCPC algorithms can be applied to tackle more effectively the high correlations that have been noted among these features [44] and uncover the underlying combinatorial code specific to a set of regulatory sequences of interest. However, before the NCPC algorithm can be used on genomewide data, technical artefacts (e.g. systematic biases in reporter assays or tested enhancers) need to removed and biases in the data accounted or corrected for, otherwise they might lead to spurious associations [45], [46].

Although we have focused on TF binding and CRM activity in this paper, our NCPC algorithms are applicable to other biological problems involving possible highly correlated features. For instance, high-throughput imaging of knock-down strains can produce large sets of highly correlated visual features describing cell shape [47]–[49]. Our NCPC algorithms can be applied to explore the relationships between these visual features and the genes knocked down, or between these features and characteristics (e.g., elongation) of the cells involved. Similarly, genome-wide RNAi screens with multiple classes of phenotypic readout [50], [51] might produce features (phenotypes) that are highly correlated, in addition to features of gene functional and spatial/temporal annotation. In the ideal case, we can find out if a phenotype is a consequence of another phenotype or any of the gene features. Dissecting direct and indirect effects in these highly correlated datasets would provide further valuable insight into the underlying biological mechanisms.

A unified interface to all causal neighbourhood/Markov blanket methods benchmarked in this paper, including the NCPC/NCPC* algorithms and the DDGraph representation, is available as the R package ddgraph, which is part of Bioconductor (http://bioconductor.org/packages/2.11/bioc/html/ddgraph.html).

## Materials and Methods

### TF binding and CRM activity data

We used the data from Supplementary Figure 8 of [18]. These data include 5 TFs previously implicated in development of mesoderm during *D. melanogaster* embryogenesis: Twist (Twi), Tinman (Tin), Myocite enhancing factor 2 (Mef2), Biniou (Bin) and Bagpipe (Bap). Their binary occupancy at 310 CRMs were measured in some or all of 5 stages, leading to 15 binding profiles. Their pairwise correlations are displayed in Figure 5. The data also contain the in vivo-tested expression patterns of the 310 CRMs. Most of these (210) did not show expression in the mesoderm, but showed expression in other tissues during embryogenesis. Out of the 100 that did show mesodermal expression, they were classified in 6 broad categories based on expression in specific tissues: Mesodermal (Meso), Mesodermal and Somatic Muscle (Meso&SM), Visceral Muscle (VM), Visceral and Somatic Muscle (VM&SM), Somatic Muscle (SM) and Cardiac Muscle (CM). We focused on the first five in our analysis, like in the original paper.

### Synthetic dataset

To construct the synthetic dataset we used Hill-climbing with BIC to infer a Bayesian network from the real biological dataset ([18]; see the previous section). We estimated the mean number of causal parents per node to be roughly 1.5 and the maximum to be 2. We therefore assumed a binomial distribution for the number of causal parents. We used a beta distribution to generate the probabilities in the conditional probability table associated with each node. With these distributions we generated a network structure that had both marginal probabilities and pairwise correlations similar to the real data. We used this network structure to generate binary data for 15 nodes in the network, which is the number of TF binding profiles in the real data. The target variable is generated separately using a noisy AND function.

To generate the CRM class target variables we considered a causal neighbourhood of size 2 and used a noisy AND function, representing the simplest combinatorial code of 2 TFs. The noise in the AND function is incorporated into both the inputs and the output of the function. The noise in the inputs models the activity of other TFs, which might, for example, inhibit the CRM activity in the presence of the TF, or activate the CRM in the absence of the TF. The noise in the output models the noise in the reporter assay used to find the activity of a CRM. Let *F*(*R_A_*, *R_B_*) be a boolean AND function with two inputs. Thus *F*(*R_A_*, *R_B_*) = 1 only if *R_A_* = *R_B_* = 1. Further, let *A* and *B* denote the real functional binding profiles of two TFs that constitute the combinatorial code. The noise at the input of the boolean AND function can be modelled by “readout” probabilities: *output* = *F*(*R_A_*, *R_B_*) · *P*(*R_A_*|*A*) · *P*(*R_B_*|*B*). If we assume that the conditional probabilities have the same distribution for *A* and *B*: *P*(*R_A_*|*A*) = *P*(*R_B_*|*B*), then we just need to specify two readout probabilities. We set these to be *P*(*R_A_* = 1|*A* = 1) = 0.5 and *P*(*R_A_* = 1|*A* = 0) = 0.1. At the output of boolean AND function, we use a false positive rate of 0.01 and false negative rate of 0.2. This parameter setting results in 10% of the CRMs being active, similar to the Zinzen et al. data. Furthermore, the data generated for these CRMs from the noisy AND function is weakly correlated (correlation between 0.17 and 0.25) with *A* and *B*. This level of correlation is also similar to the observed correlations between the CRM classes and TF binding profiles in the real data.

To incorporate the two scenarios “Time” and “Hidden” described in the main text, we randomly chose three variables in each simulated network. We then rewired these three variables to match each scenario. For the “Time” scenario we allowed for the first variable to have causal parents as in the unmodified network, while variables two and three have causal parents only from the scenario. However, they retained the original causal children of the unmodified network. This ensured that we can fully control the correlation between these three variables, but also leave it as much as possible in the context of rest of the network. In the “Hidden” scenario, we generated an additional hidden variable and made it a causal parent for the three variables in the scenario. Now the three variables only retained their original causal children, but not their causal parents. To generate the binary profile of the target variable, we applied the noisy AND function as before.

The hill-climbing and IAMB algorithms were applied using the bnlearn R package, and PC algorithm was applied using the pcalg R package. Both can be accessed using a unified interface in our R package ddgraph.

### Applying NCPC and other algorithms and assessing their performance

For NCPC and NCPC* we used the Monte-Carlo chi-square test, while for the IAMB algorithms we used the Mutual Information test recommended by the authors [28], but with Monte Carlo-calculated P-values due to small sample sizes. We compared these two tests in a simple case of two variables and found that the Monte-Carlo chisquare test was slightly better than the Monte-Carlo Mutual Information test. However, their differences were not noticeable when applied to our synthetic data. For MMHC we use the default constraint-based algorithm (MMPC).

To assess the performance of the algorithms, we defined a prediction as correct if there are no false positive and no false negatives. The accuracy was measured by the prediction rate, which was the proportion of correct predictions over all the synthetic networks. We also defined precision as TP/(TP+FP), where TP is the number of true positives, and FP is the number of false positives. Additionally, we defined recall as TP/(TP+FN) where FN is the number of false negatives. Rates of precision and recall were also averaged over all the synthetic networks.

### Controlling the power of conditional independence tests in the NCPC algorithms

As the size of the conditioning set increases, the power of the test decreases. To increase power, we limited the total count *l* of datapoints per conditioning set to 10. Our NCPC and NCPC* algorithms performed the test if this requirement was met and considered the variables to be dependent otherwise.

Alternatively, one may constrain the size of the conditioning set. Since our data are binary, we set the maximal size of the conditioning set *k* to 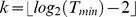, where *T_min_* is the smaller of the number of ones and the number of zeros in *T*. We found that these two rules performed similarly on our binary data. The second rule, however, may also be applied to continuous features with a binary target variable.

### Testing enrichment of TF combinations

For *n* TFs, each of which is either present or not at a CRM, we performed Fisher's exact test to test whether a combination of presence and absence of these TFs is statistically significantly associated with a CRM class. This test essentially compares the frequencies of the combination within this CRM class and across the other four classes. We applied the Benjamini-Hochberg correction [19], which adjusts the P-values to control the False Discovery Rate (FDR), and retained those combinations with adjusted P-values smaller than 0.15.

## Supporting Information

Text S1 **Supplementary information.** Contains Supplementary and Supplementary Figures S1–15. The Supplementary Text S1 contains a proof of mathematical inconsistency of the joint and conditional joint dependence patterns, the description of the PC algorithm and a detailed pseudo-code of the NCPC algorithms.
(PDF)
