# Supplementary material for: A Graphical Modelling Approach to the Dissection of Highly Correlated Transcription Factor Binding Site Profiles
Source: PLoS Comput Biol. 2012 Nov 8;8(11):e1002725. doi: 10.1371/journal.pcbi.1002725 (PMC3493460; doi:10.1371/journal.pcbi.1002725)
Supplement: Text S1 — Supplementary information. Contains Supplementary and Supplementary Figures S1–15. The Supplementary Text S1 contains a proof of mathematical inconsistency of the joint and conditional joint dependence patterns, the description of the PC algorithm and a detailed pseudo-code of the NCPC algorithms. (PDF) [file pcbi.1002725.s001.pdf]

Supplementary Information

Supplementary Text S1

Joint and conditional joint dependence patterns of random variables

In this section we prove that no discrete random variables can have the joint and conditional joint dependence patterns of conditional independence (Candidate Patterns 1 and 2).

Theorem 1. *There are no such discrete random variables A, B, T for which the following set of dependencies are all simultaneously true:*

$$A \not\perp\!\!\!\perp T \tag{1}$$

$$B \not\perp\!\!\!\perp T \tag{2}$$

$$A \perp\!\!\!\perp T \mid B \tag{3}$$

$$B \perp\!\!\!\perp T \mid A \tag{4}$$

When all of $P(A|B), P(B|A), P(A), P(B)$ have non-zero probabilities.

Proof. We will prove this theorem by contradiction. We start by assuming all four Equations 1 - 4 are true. In that case, the joint probability $P(A, B, T)$ can be written as:

$$P(A, B, T) = P(A, T|B)P(B) = P(B, T|A)P(A) \tag{5}$$

Given Equations 3 and 4 we can rewrite this as:

$$P(A|B)P(T|B)P(B) = P(B|A)P(T|A)P(A) \tag{6}$$

Now we use the property that $P(A|B), P(B|A), P(A), P(B)$, and thus $P(A, B)$ have non-zero probabilities. When these are zero, the system reduces to trivial cases where either A and B are identical, or one of them is a constant.

We divide both sides of the equation by $P(A, B)$ to obtain:

$$P(T|B) = P(T|A) \tag{7}$$

We can repeat this derivation for various values of random variables A, B, T, and for any of these values we would arrive to the same equation. Without loss of generality assume that A, B, T are binary. In that case for $A = 1, B = 1, T = 1$ and $A = 0, B = 1, T = 1$ we get:

$$P(T = 1|B = 1) = P(T = 1|A = 1) \tag{8}$$

$$P(T = 1|B = 1) = P(T = 1|A = 0) \tag{9}$$

This implies that $P(T = 1|A = 0) = P(T = 1|A = 1)$ which in turns implies that A and T are marginally independent when $T = 1$. Same reasoning can be applied to the case when $T = 0$, thus showing that A and T are marginally independent which contradicts the initial assumption that they are dependent (Equation 1). Similar argument can be made for discrete variables with more than two values as well. □

The same theorem can be proven for conditional joint pattern of dependence. Both the formulation and the proof are equivalent, but conditional on a set of variables.

We conjecture that the same theorem and reasoning might apply for continuous random variables as well.

Constraint-based algorithms

Conditional independence can be established at least in two ways: (i) by performing conditional independence tests for subsets of variables, the approach taken by constraint-based network inference algorithms; and (ii) by building a Bayesian Network for all the variables, the approach taken by score-based algorithms.

The PC algorithm is one of the first algorithms proposed for inference of Bayesian networks [15]. It is constraint-based and builds a Bayesian network in two steps. In the first step (PC-skeleton), the algorithm starts with a fully connected graph and then performs conditional independence tests of increasing order to delete edges. This recovers the structure of direct and indirect dependencies, that is, the undirected network skeleton. In the second step, edges are oriented using a set of rules.

Our algorithm uses a procedure similar to the PC-skeleton algorithm to distinguish direct from indirect dependencies. However, it does not perform the second step as we are not interested in inferring causation. For completeness, below we introduce the PC skeleton algorithm. With $Adj(X)$ we denote the nodes in a graph that have an edge to X, i.e. adjacent nodes in a graph.

PC-skeleton algorithm

Input: Dataset $\mathcal{D}$

1. Let $n = 0$.
2. Let $\mathcal{G}$ be a complete undirected graph with nodes representing variable from $\mathcal{D}$.
3. Repeat:
   (a) For all pairs of variables (X, Y) connected with an edge in $\mathcal{D}$ test if they are conditionally independent given a set of variables $\mathbf{S}$ of size n, where $\mathbf{S} \subset Adj(X)$ or $\mathbf{S} \subset Adj(Y)$. If they are conditionally independent, remove the edge $X - Y$ from the graph.
   (b) Set $n = n + 1$ until no variable has greater than n adjacencies,or a stopping condition is satisfied.
4. Return $\mathcal{G}$

The algorithm assumes the conditional independence test giving reliable results. In practice, a conditional independence test, like a chi-square test, is used with a fixed P-value cut-off α (typically set to $\alpha = 0.05$). This cut-off specifies the Type I error (false positive) rate under the null hypothesis. The Type II error (false negative) rate of conditional independence tests is harder to control for. This rate will depend on sample size, size of the conditioning test, size of effects we wish to detect and the α value. Either the power can be estimated taking into account these four factors, or the maximal size of the conditioning set (or data point per conditioning set) can be capped [14]. The Type II error rate increases as the size of the conditioning set $\mathbf{S}$ increases, that is, the test looses power as we condition on more variables.

More recently, a framework for general local learning (GLL) was proposed [13]. Instead of systematically doing tests of increasing order, the Markov blanket for each of the nodes is found separately, and then a network constructed by violating the smallest number of inferred Markov blankets. These algorithms have a forward and backward phase, in the forward variables are added to the tentative Markov blankets of a node, and in the backward conditional independence tests are used to remove false positives from the tentative Markov blankets. The advantage of such an approach is that it separately finds the best local Markov blanket for each of the nodes, and is thus less sensitive to error propagation through the network inference steps, like it is the case in the PC algorithm.

Detailed description of NCPC and NCPC* algorithms

Below we give a more detailed pseudo-code for the NCPC and NCPC* algorithms. A conditional independence test I_α return either true if the P-value of the test is greater than α or false otherwise.

NCPC algorithm

Input:

- Matrix X with columns representing different variables $(X_1, X_2, ...X_m)$ and rows representing observations.
- Column vector T of target variable values, with observations corresponding to those of X.
- Conditional independence test I_α (with a given α value threshold and optionally: a minimal number of observations per set of values of in a conditioning set l).
- (Optionally) Maximal size of conditioning set k.

Algorithm:

1. Initialise a list of candidates of variables for direct dependence: $\mathbf{C} = \{X_i : I_\alpha(X_i, T) = \text{false}\}$
2. Let $n = 1$
3. Repeat:
   (a) Enumerate all subsets $\mathbf{S_n} : \{\mathbf{S} : \mathbf{S} \subset \mathbf{C} \ \wedge |\mathbf{S}| = n\}$
   (b) For each $X_i \in \mathbf{C}$ and $\mathbf{S} \in \mathbf{S_n}$ ($X_i \notin \mathbf{S}$) test $I_\alpha(X_i, T|\mathbf{S})$ and save the result
   (c) For each $X_i \in \mathbf{C}$ check if there is at least one $\mathbf{S}$ for which $I_\alpha(X_i, T|\mathbf{S}) = \text{true}$. If such $\mathbf{S}$ exists, remove X_i from list of candidates $\mathbf{C}$.
   (d) Set $n = n + 1$.
   (e) Break out of the loop if $n > k$ or if $n \geq |\mathbf{C}|$
4. Mark all remaining variables in $\mathbf{C}$ as having *direct* dependence
5. Mark all variables removed in Step 3. as having *indirect* dependence
6. For each pair of variables X_i, X_j marked to have indirect dependence, examine all pairs of tests performed in Step 3. If a test pair is of form: $I_\alpha(X_i, T|\{X_j, \mathbf{S}'\}) = \text{true}$ and $I_\alpha(X_j, T|\{X_i, \mathbf{S}'\}) = \text{true}$ then mark this test pair as inconsistent.
7. If a variable X_i is removed only using inconsistent tests, then mark it as having *joint* dependence
8. Mark all remaining variables without any calls as having *no* dependence
9. Return calls for each of the variables in X

NCPC* algorithm

Input: (same as for NCPC algorithm)
Algorithm:

1. Initialise a list of candidates of variables for direct dependence with ordered pairs: $\mathbf{C_d} = \{(X_i, \varnothing) : I_\alpha(X_i, T) = \text{false}\}$
2. Extend a list of candidate variables with ordered pairs (conditional dependent variable, conditioning set): $\mathbf{C} = \mathbf{C_d} \cup \{(X_j, X_i) : I_\alpha(X_j, T|X_i) = \text{false} \wedge X_i \in \mathbf{C_d}\}$
3. Let $n = 1$
4. Repeat:
   (a) Enumerate all subsets $\mathbf{S_n} : \{\mathbf{S} : \mathbf{S} \subset \mathbf{C} \ \wedge ||\mathbf{S}|| = n\}$. Since $\mathbf{S}$ consists of ordered pairs, $||\mathbf{S}||$ measure the number of non-empty values in all pairs. E.g. $||\{(X_i, \varnothing), (X_j, X_k)|| = 3$.
   (b) For each ordered pair $(X_i, X_j) \in \mathbf{C}$ (X_j can be $\varnothing$) and $\mathbf{S} \in \mathbf{S_n}$ ($X_i \notin \mathbf{S}$) test $I_\alpha(X_i, T|\{X_j, \mathbf{S}\})$ and save the result
   (c) For each $(X_i, X_j) \in \mathbf{C}$ check if there is at least one $\mathbf{S}$ for which $I_\alpha(X_i, T|\{X_j, \mathbf{S}\}) = \text{true}$. If such $\mathbf{S}$ exists, remove (X_i, X_j) from list of candidates $\mathbf{C}$, together with all those ordered pairs where X_i is in the second place in the pair.
   (d) Set $n = n + 1$.

(e) Break out of the loop if $n > k$ or if $n \geq \|\mathbf{C}\|$

5. Mark all remaining pairs $(X_i, X_j) \in \mathbf{C}$ as having *direct* dependence if $X_j = \varnothing$ and *conditional* dependence otherwise
6. Mark all pairs $(X_i, \varnothing)$ removed in Step 3. as having *indirect* dependence
7. For each pair of ordered pairs variables $(X_i, X_j), (X_p, X_r)$ removed in Step 3, examine all tests performed in Step 3. If a test pair is of form: $I_\alpha(X_i, T|\{X_p, X_j, \mathbf{S}'\}) =$ true and $I_\alpha(X_p, T|\{X_i, X_r, \mathbf{S}'\}) =$ true then mark this test pair as inconsistent.
8. If an ordered pair (X_i, X_j) is removed only using inconsistent tests, then mark it as having *joint* dependence if $X_j = \varnothing$ and *conditional joint* dependence otherwise
9. Mark all remaining variables without any calls as having *no* dependence
10. Return calls for each of the variables in X

We also implemented NCPC and NCPC* with multiple testing correction as suggested by [21] for the PC algorithm. Briefly, at each iteration of step 3 in NCPC (step 4 in NCPC*), and for each candidate retained, a list is kept of P-values from conditional independence tests given other candidates **C**. The maximum of these P-values is used as the candidate P-value and multiple testing correction applied to these maximal P-values over all candidates. At a predefined false discovery rate (FDR), candidates with adjusted P-values above the FDR threshold are removed from the candidate set. This procedure controls the false discovery rate of edges with direct dependence. Either the Benjamini-Hochberg [19] for independent tests or Benjamini-Yekutieli correction [20] for dependent tests can be used.

Supplementary Figure S1-S15

Algorithm	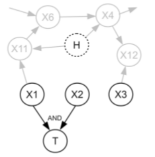 Correlation = 0	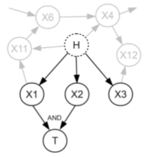 Correlation = 0.25	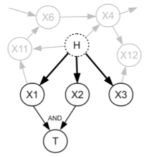 Correlation = 0.50	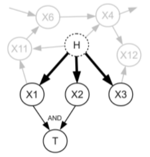 Correlation = 0.75
		Number of data points = 300		
NCPC dir	**0.467 ± 0.031**	**0.422 ± 0.031**	0.183 ± 0.024	0.002 ± 0.003
NCPC dir+jnt	**0.480 ± 0.031**	**0.461 ± 0.031**	**0.268 ± 0.027**	**0.294 ± 0.028**
NCPC* dir	0.408 ± 0.030	0.316 ± 0.029	0.114 ± 0.020	0.001 ± 0.002
NCPC* dir+jnt	**0.472 ± 0.031**	**0.438 ± 0.031**	**0.260 ± 0.027**	**0.308 ± 0.029**
NCPC* dir+cond	0.367 ± 0.030	0.280 ± 0.028	0.099 ± 0.019	0.000 ± 0.000
NCPC* dir+jnt+cond	**0.420 ± 0.031**	0.374 ± 0.030	**0.241 ± 0.027**	**0.307 ± 0.029**
NCPC* dir+jnt+cond+cjnt	0.350 ± 0.030	0.277 ± 0.028	0.192 ± 0.024	**0.306 ± 0.029**
PC algorithm	0.381 ± 0.030	0.124 ± 0.020	0.018 ± 0.008	0.003 ± 0.003
Hill-climbing with BIC	**0.483 ± 0.031**	0.155 ± 0.022	0.032 ± 0.011	0.000 ± 0.000
Hill-climbing with BDe	0.122 ± 0.020	0.172 ± 0.023	0.100 ± 0.019	0.017 ± 0.008
IAMB	0.359 ± 0.030	0.348 ± 0.030	**0.208 ± 0.025**	0.074 ± 0.016
FastIAMB	0.319 ± 0.029	0.286 ± 0.028	0.167 ± 0.023	0.073 ± 0.016
InterIAMB	0.355 ± 0.030	0.356 ± 0.030	**0.209 ± 0.025**	0.083 ± 0.017
MMPC	0.380 ± 0.030	0.252 ± 0.027	0.066 ± 0.015	0.010 ± 0.006
MMHC with BIC	0.354 ± 0.030	0.103 ± 0.019	0.007 ± 0.005	0.000 ± 0.000
MMHC with BDe	0.358 ± 0.030	0.119 ± 0.020	0.017 ± 0.008	0.000 ± 0.000
		Number of data points = 500		
NCPC dir	0.723 ± 0.028	**0.711 ± 0.028**	**0.487 ± 0.031**	0.056 ± 0.014
NCPC dir+jnt	0.704 ± 0.028	**0.695 ± 0.029**	**0.466 ± 0.031**	**0.299 ± 0.028**
NCPC* dir	0.676 ± 0.029	0.607 ± 0.030	0.330 ± 0.029	0.032 ± 0.011
NCPC* dir+jnt	0.712 ± 0.028	**0.698 ± 0.028**	**0.451 ± 0.031**	**0.294 ± 0.028**
NCPC* dir+cond	0.549 ± 0.031	0.497 ± 0.031	0.287 ± 0.028	0.040 ± 0.012
NCPC* dir+jnt+cond	0.570 ± 0.031	0.577 ± 0.031	0.404 ± 0.030	**0.299 ± 0.028**
NCPC* dir+jnt+cond+cjnt	0.481 ± 0.031	0.457 ± 0.031	0.288 ± 0.028	**0.289 ± 0.028**
PC algorithm	0.610 ± 0.030	0.131 ± 0.021	0.019 ± 0.008	0.007 ± 0.005
Hill-climbing with BIC	**0.789 ± 0.025**	0.402 ± 0.030	0.115 ± 0.020	0.006 ± 0.005
Hill-climbing with BDe	0.269 ± 0.027	0.363 ± 0.030	0.296 ± 0.028	0.083 ± 0.017
IAMB	0.512 ± 0.031	0.496 ± 0.031	0.352 ± 0.030	0.119 ± 0.020
FastIAMB	0.455 ± 0.031	0.425 ± 0.031	0.302 ± 0.028	0.118 ± 0.020
InterIAMB	0.501 ± 0.031	0.504 ± 0.031	0.358 ± 0.030	0.135 ± 0.021
MMPC	0.626 ± 0.030	0.389 ± 0.030	0.130 ± 0.021	0.014 ± 0.007
MMHC with BIC	0.620 ± 0.030	0.204 ± 0.025	0.019 ± 0.008	0.000 ± 0.000
MMHC with BDe	0.577 ± 0.031	0.205 ± 0.025	0.036 ± 0.012	0.000 ± 0.000
		Number of data points = 1000		
NCPC dir	0.876 ± 0.020	**0.881 ± 0.020**	**0.841 ± 0.023**	**0.400 ± 0.030**
NCPC dir+jnt	0.832 ± 0.023	0.826 ± 0.024	**0.808 ± 0.024**	**0.403 ± 0.030**
NCPC* dir	**0.884 ± 0.020**	**0.860 ± 0.022**	0.770 ± 0.026	0.218 ± 0.026
NCPC* dir+jnt	0.847 ± 0.022	**0.835 ± 0.023**	**0.817 ± 0.024**	**0.362 ± 0.030**
NCPC* dir+cond	0.706 ± 0.028	0.682 ± 0.029	0.615 ± 0.030	0.170 ± 0.023
NCPC* dir+jnt+cond	0.693 ± 0.029	0.675 ± 0.029	0.685 ± 0.029	0.310 ± 0.029
NCPC* dir+jnt+cond+cjnt	0.618 ± 0.030	0.589 ± 0.031	0.550 ± 0.031	0.195 ± 0.025
PC algorithm	0.797 ± 0.025	0.153 ± 0.022	0.058 ± 0.014	0.010 ± 0.006
Hill-climbing with BIC	**0.923 ± 0.017**	0.826 ± 0.024	0.651 ± 0.030	0.051 ± 0.014
Hill-climbing with BDe	0.470 ± 0.031	0.636 ± 0.030	0.650 ± 0.030	**0.358 ± 0.030**
IAMB	0.561 ± 0.031	0.546 ± 0.031	0.536 ± 0.031	0.299 ± 0.028
FastIAMB	0.520 ± 0.031	0.451 ± 0.031	0.450 ± 0.031	0.263 ± 0.027
InterIAMB	0.561 ± 0.031	0.550 ± 0.031	0.539 ± 0.031	0.326 ± 0.029
MMPC	0.762 ± 0.026	0.594 ± 0.030	0.339 ± 0.029	0.068 ± 0.016
MMHC with BIC	0.842 ± 0.023	0.436 ± 0.031	0.125 ± 0.021	0.002 ± 0.003
MMHC with BDe	0.741 ± 0.027	0.379 ± 0.030	0.129 ± 0.021	0.007 ± 0.005

Supplementary Figure S1. Proportion of correct predictions for the "Hidden" scenario. Results in the same format as Figure 4 but for the scenario "Hidden".

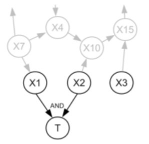

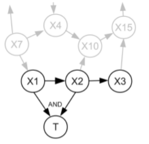

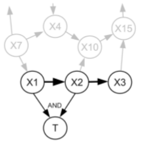

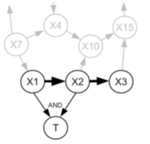

	Correlation = 0		Correlation = 0.25		Correlation = 0.50		Correlation = 0.75	
Algorithm	Precision	Recall	Precision	Recall	Precision	Recall	Precision	Recall
Number of data points = 300								
NCPC dir	**0.87**	0.50	**0.92**	0.56	**0.89**	0.48	0.81	0.26
NCPC dir+jnt	0.68	0.66	0.75	**0.71**	0.77	**0.66**	0.78	**0.72**
NCPC* dir	**0.89**	0.47	**0.93**	0.50	**0.89**	0.41	0.82	0.23
NCPC* dir+jnt	0.69	0.66	0.75	0.69	0.77	**0.65**	0.78	**0.71**
NCPC* dir+cond	0.74	0.52	0.76	0.52	0.74	0.43	0.63	0.24
NCPC* dir+jnt+cond	0.64	0.69	0.67	**0.70**	0.70	**0.66**	0.72	**0.71**
NCPC* dir+jnt+cond+cjnt	0.59	0.70	0.61	**0.71**	0.63	**0.66**	0.68	**0.72**
PC algorithm	0.74	0.42	0.56	0.44	0.59	0.25	0.45	0.10
Hill-climbing with BIC	0.82	0.56	0.83	0.53	**0.86**	0.48	**0.89**	0.47
Hill-climbing with BDe	0.39	**0.77**	0.39	**0.73**	0.39	**0.68**	0.37	0.62
IAMB	0.62	0.65	0.63	0.68	0.62	0.62	0.57	0.53
FastIAMB	0.57	0.62	0.56	0.63	0.53	0.58	0.50	0.51
InterIAMB	0.62	0.65	0.63	0.68	0.62	0.62	0.57	0.53
MMPC	0.82	0.48	0.84	0.44	0.71	0.26	0.54	0.14
MMHC with BIC	**0.90**	0.38	**0.89**	0.36	0.79	0.19	0.51	0.07
MMHC with BDe	0.77	0.43	0.76	0.39	0.67	0.21	0.40	0.07
Number of data points = 500								
NCPC dir	**0.92**	0.69	**0.93**	0.77	**0.94**	0.77	**0.88**	0.48
NCPC dir+jnt	0.76	**0.84**	0.82	**0.85**	0.87	**0.81**	0.83	0.66
NCPC* dir	**0.94**	0.63	**0.95**	0.65	**0.95**	0.59	**0.90**	0.40
NCPC* dir+jnt	0.76	0.83	0.83	**0.83**	0.87	**0.79**	0.84	0.64
NCPC* dir+cond	0.84	0.66	0.83	0.67	0.83	0.60	0.73	0.41
NCPC* dir+jnt+cond	0.71	**0.84**	0.77	**0.84**	0.79	**0.79**	0.74	0.64
NCPC* dir+jnt+cond+cjnt	0.65	**0.85**	0.67	**0.85**	0.66	**0.80**	0.66	0.66
PC algorithm	0.78	0.56	0.58	0.61	0.64	0.42	0.56	0.18
Hill-climbing with BIC	0.88	0.72	0.88	0.64	**0.92**	0.57	**0.92**	0.50
Hill-climbing with BDe	0.48	**0.88**	0.49	**0.84**	0.50	**0.81**	0.45	**0.71**
IAMB	0.69	0.83	0.68	**0.81**	0.69	**0.79**	0.61	0.63
FastIAMB	0.62	0.78	0.61	0.77	0.59	0.73	0.53	0.60
InterIAMB	0.68	0.82	0.69	**0.81**	0.69	**0.79**	0.62	0.64
MMPC	0.87	0.60	0.86	0.56	0.82	0.41	0.68	0.23
MMHC with BIC	**0.93**	0.51	0.89	0.48	0.87	0.31	0.67	0.14
MMHC with BDe	0.82	0.56	0.78	0.51	0.75	0.33	0.54	0.15
Number of data points = 1000								
NCPC dir	**0.95**	0.92	**0.95**	**0.94**	**0.94**	**0.92**	**0.94**	0.81
NCPC dir+jnt	0.88	**0.97**	0.89	**0.97**	0.91	**0.95**	0.90	**0.84**
NCPC* dir	**0.97**	0.87	**0.98**	0.88	**0.97**	0.82	**0.95**	0.62
NCPC* dir+jnt	0.89	**0.97**	0.90	**0.97**	0.92	**0.94**	0.91	**0.82**
NCPC* dir+cond	0.88	0.89	0.88	0.88	0.87	0.82	0.82	0.62
NCPC* dir+jnt+cond	0.83	**0.97**	0.83	**0.97**	0.85	**0.94**	0.83	**0.82**
NCPC* dir+jnt+cond+cjnt	0.76	**0.98**	0.75	**0.97**	0.74	**0.95**	0.67	**0.82**
PC algorithm	0.86	0.80	0.59	0.80	0.70	0.64	0.68	0.37
Hill-climbing with BIC	**0.95**	0.93	**0.97**	0.89	**0.97**	0.80	**0.96**	0.57
Hill-climbing with BDe	0.61	**0.97**	0.67	**0.96**	0.66	**0.94**	0.62	**0.85**
IAMB	0.72	**0.97**	0.73	**0.95**	0.72	**0.93**	0.67	**0.81**
FastIAMB	0.67	0.92	0.66	0.93	0.65	0.89	0.59	0.77
InterIAMB	0.74	**0.97**	0.74	**0.96**	0.74	**0.93**	0.70	**0.83**
MMPC	0.91	0.83	0.91	0.77	0.89	0.65	0.81	0.44
MMHC with BIC	**0.96**	0.76	**0.94**	0.67	**0.95**	0.54	0.88	0.30
MMHC with BDe	0.86	0.77	0.84	0.69	0.87	0.57	0.74	0.33

Supplementary Figure S2. Precision and recall for the "Time" scenario. Precision and recall for the "Time" scenario. Precision is measured as TP/(TP+FP) and recall as TP/(TP+FN) where TP is the number of true positives, FP number of false positives and FN number of false negatives. The best precision and recall rates are shown in bold together with other values that are not significantly different (where the difference is smaller than 0.04 which is roughly two times the 95% confidence interval for these values)

	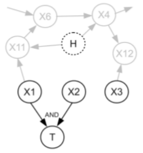		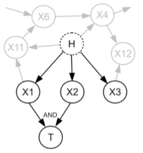		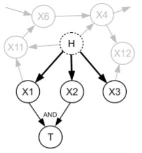		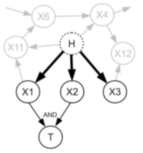	
	Correlation = 0		Correlation = 0.25		Correlation = 0.50		Correlation = 0.75	
Algorithm	Precision	Recall	Precision	Recall	Precision	Recall	Precision	Recall
Number of data points = 300								
NCPC dir	**0.90**	0.71	**0.93**	0.66	**0.88**	0.45	0.74	0.14
NCPC dir+jnt	0.80	0.80	0.82	**0.78**	0.76	**0.68**	0.72	**0.76**
NCPC* dir	**0.93**	0.67	**0.94**	0.59	**0.88**	0.40	0.75	0.13
NCPC* dir+jnt	0.80	0.79	0.82	**0.76**	0.76	**0.66**	0.72	**0.76**
NCPC* dir+cond	0.82	0.70	0.82	0.62	0.74	0.41	0.61	0.14
NCPC* dir+jnt+cond	0.75	0.81	0.75	**0.77**	0.70	**0.67**	0.70	**0.77**
NCPC* dir+jnt+cond+cjnt	0.70	0.82	0.68	**0.78**	0.65	**0.68**	0.69	**0.77**
PC algorithm	0.86	0.64	0.60	0.47	0.47	0.14	0.29	0.05
Hill-climbing with BIC	0.87	0.75	0.85	0.59	**0.87**	0.48	**0.84**	0.44
Hill-climbing with BDe	0.41	**0.87**	0.46	**0.76**	0.44	0.60	0.38	0.49
IAMB	0.69	0.80	0.69	**0.78**	0.64	**0.65**	0.55	0.51
FastIAMB	0.65	0.76	0.61	0.72	0.54	0.60	0.48	0.47
InterIAMB	0.69	0.79	0.70	**0.78**	0.63	**0.65**	0.56	0.51
MMPC	0.85	0.69	0.85	0.52	0.70	0.24	0.43	0.08
MMHC with BIC	**0.93**	0.62	0.87	0.43	0.68	0.13	0.37	0.03
MMHC with BDe	0.83	0.68	0.76	0.46	0.56	0.14	0.25	0.04
Number of data points = 500								
NCPC dir	**0.94**	0.88	**0.95**	0.87	**0.92**	0.73	0.83	0.31
NCPC dir+jnt	0.86	**0.95**	0.87	**0.93**	0.83	**0.80**	0.80	**0.71**
NCPC* dir	**0.96**	0.84	**0.97**	0.80	**0.94**	0.62	0.83	0.27
NCPC* dir+jnt	0.87	**0.95**	0.88	**0.93**	0.84	**0.78**	0.80	**0.70**
NCPC* dir+cond	0.86	0.86	0.87	0.82	0.81	0.63	0.67	0.28
NCPC* dir+jnt+cond	0.80	**0.95**	0.82	**0.93**	0.76	**0.78**	0.74	**0.71**
NCPC* dir+jnt+cond+cjnt	0.73	**0.96**	0.74	**0.93**	0.68	**0.79**	0.71	**0.72**
PC algorithm	0.91	0.81	0.55	0.63	0.53	0.25	0.39	0.08
Hill-climbing with BIC	**0.93**	**0.92**	0.88	0.75	**0.92**	0.55	**0.91**	0.47
Hill-climbing with BDe	0.51	**0.96**	0.59	**0.90**	0.57	**0.78**	0.48	0.59
IAMB	0.75	**0.94**	0.75	**0.93**	0.69	**0.81**	0.61	0.58
FastIAMB	0.69	0.90	0.68	0.87	0.62	**0.77**	0.51	0.54
InterIAMB	0.74	**0.94**	0.76	**0.93**	0.69	**0.81**	0.62	0.59
MMPC	0.91	0.84	0.89	0.66	0.80	0.37	0.58	0.14
MMHC with BIC	**0.97**	0.80	0.89	0.55	0.83	0.22	0.44	0.05
MMHC with BDe	0.89	0.83	0.76	0.60	0.70	0.23	0.33	0.05
Number of data points = 1000								
NCPC dir	**0.95**	**0.98**	**0.95**	**0.97**	**0.96**	**0.95**	0.92	0.67
NCPC dir+jnt	0.91	**0.99**	0.90	**0.99**	0.90	**0.98**	0.86	0.75
NCPC* dir	**0.97**	**0.96**	**0.97**	0.95	**0.98**	0.90	**0.93**	0.54
NCPC* dir+jnt	0.92	**0.99**	0.91	**0.99**	0.91	**0.98**	0.86	0.72
NCPC* dir+cond	0.88	**0.97**	0.88	0.96	0.89	0.90	0.79	0.54
NCPC* dir+jnt+cond	0.85	**0.99**	0.84	**0.99**	0.85	**0.98**	0.77	0.72
NCPC* dir+jnt+cond+cjnt	0.80	**1.00**	0.78	**0.99**	0.77	**0.98**	0.67	0.74
PC algorithm	**0.94**	0.94	0.52	0.83	0.52	0.52	0.51	0.16
Hill-climbing with BIC	**0.96**	**0.98**	0.93	**0.96**	**0.98**	0.83	**0.96**	0.52
Hill-climbing with BDe	0.64	**0.99**	0.75	**0.98**	0.75	**0.97**	0.68	**0.78**
IAMB	0.76	**1.00**	0.76	**1.00**	0.73	**0.97**	0.66	**0.78**
FastIAMB	0.72	**0.97**	0.69	**0.96**	0.67	0.93	0.60	0.75
InterIAMB	0.76	**1.00**	0.76	**1.00**	0.75	**0.97**	0.69	**0.80**
MMPC	0.92	0.94	0.90	0.83	0.87	0.61	0.73	0.27
MMHC with BIC	**0.98**	0.93	0.89	0.73	0.92	0.38	0.66	0.10
MMHC with BDe	0.90	0.93	0.79	0.74	0.81	0.39	0.49	0.11

Supplementary Figure S3. Precision and recall for the ”Hidden” scenario. Precision and recall for the ”Hidden” scenario. The format of this table is the same as that of Figure S2.

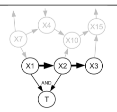

	Correlation = 0.75		
Algorithm	Correct	Precision	Recall
Number of data points = 500			
NCPC dir	0.017 ± 0.008	**0.95**	0.20
NCPC dir+jnt	**0.491 ± 0.031**	0.91	**0.79**
NCPC* dir	0.009 ± 0.006	**0.95**	0.20
NCPC* dir+jnt	**0.483 ± 0.031**	0.90	**0.78**
NCPC* dir+cond	0.013 ± 0.007	0.86	0.20
NCPC* dir+jnt+cond	**0.485 ± 0.031**	0.88	**0.78**
NCPC* dir+jnt+cond+cjnt	**0.479 ± 0.031**	0.87	**0.79**
PC algorithm	0.004 ± 0.004	0.61	0.05
Hill-climbing with BIC	0.016 ± 0.008	**0.94**	0.50
Hill-climbing with BDe	0.060 ± 0.015	0.33	**0.75**
IAMB	0.071 ± 0.016	0.83	0.53
FastIAMB	0.083 ± 0.017	0.77	0.52
InterIAMB	0.094 ± 0.018	0.82	0.54
MMPC	0.027 ± 0.010	0.69	0.23
MMHC with BIC	0.004 ± 0.004	0.72	0.14
MMHC with BDe	0.014 ± 0.007	0.57	0.15

Supplementary Figure S4. Results in the "Time" scenario with α = 0.01. Correct predictions, precision and recall in the "Time" scenario but by using α = 0.01 P-value cut-off. When we use this cut-off we recover the expected high performance of the NCPC algorithm in the most correlated case. The best values and those not significantly different are shown in bold.

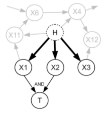

	Correlation = 0.75		
Algorithm	Correct	Precision	Recall
Number of data points = 500			
NCPC dir	0.003 ± 0.003	**0.92**	0.10
NCPC dir+jnt	**0.447 ± 0.031**	0.80	**0.86**
NCPC* dir	0.001 ± 0.002	**0.92**	0.10
NCPC* dir+jnt	**0.438 ± 0.031**	0.80	**0.86**
NCPC* dir+cond	0.003 ± 0.003	0.85	0.10
NCPC* dir+jnt+cond	**0.440 ± 0.031**	0.79	**0.86**
NCPC* dir+jnt+cond+cjnt	**0.439 ± 0.031**	0.79	**0.86**
PC algorithm	0.004 ± 0.004	0.46	0.03
Hill-climbing with BIC	0.003 ± 0.003	**0.91**	0.48
Hill-climbing with BDe	0.033 ± 0.011	0.38	0.58
IAMB	0.061 ± 0.015	0.82	0.50
FastIAMB	0.064 ± 0.015	0.75	0.48
InterIAMB	0.081 ± 0.017	0.84	0.51
MMPC	0.016 ± 0.008	0.58	0.13
MMHC with BIC	0.000 ± 0.000	0.48	0.04
MMHC with BDe	0.001 ± 0.002	0.33	0.05

Supplementary Figure S5. Results in the "Hidden" scenario with α = 0.01. Correct predictions, precision and recall in the "Hidden" scenario but by using α = 0.01 P-value cut-off. When we use this cut-off we recover the expected high performance of the NCPC algorithm in the most correlated case. The best values and those not significantly different are shown in bold.

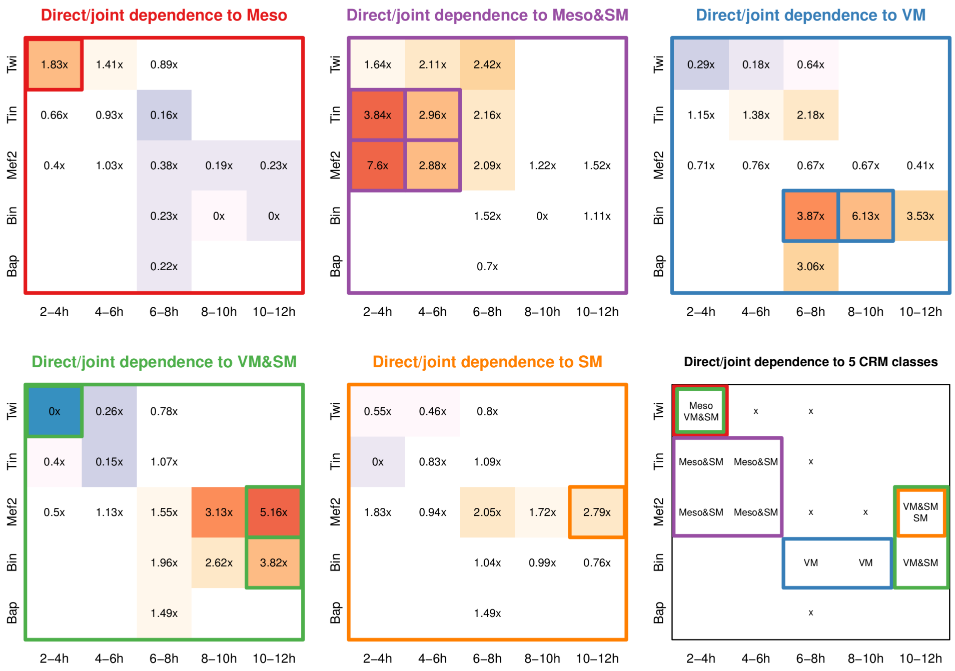

Supplementary Figure S6. Causal neighbourhood marked with rectangles and marginal dependence colour-coded for the mesodermal dataset. The layout of these plots is same to that of Figure 4 of the original paper where the data has been first published [18]. The colour-coding of *marginal* dependence is same as in Figure 6. The numbers represent fold difference for binding of TFs in each of the classes. For instance, in the "Meso" CRMs, Twi 2-4h is bound 1.83 times more frequently than in the rest of the CRMs. With solid rectangles we mark those combinations of TFs and time points that are in the causal neighbourhood of a CRM class (direct or joint dependence). The last figure summarizes the causal neighbourhoods for the 5 CRM classes. With "x" we mark those TF/time interval combinations which are not in causal neighbourhood of any of the CRM classes.

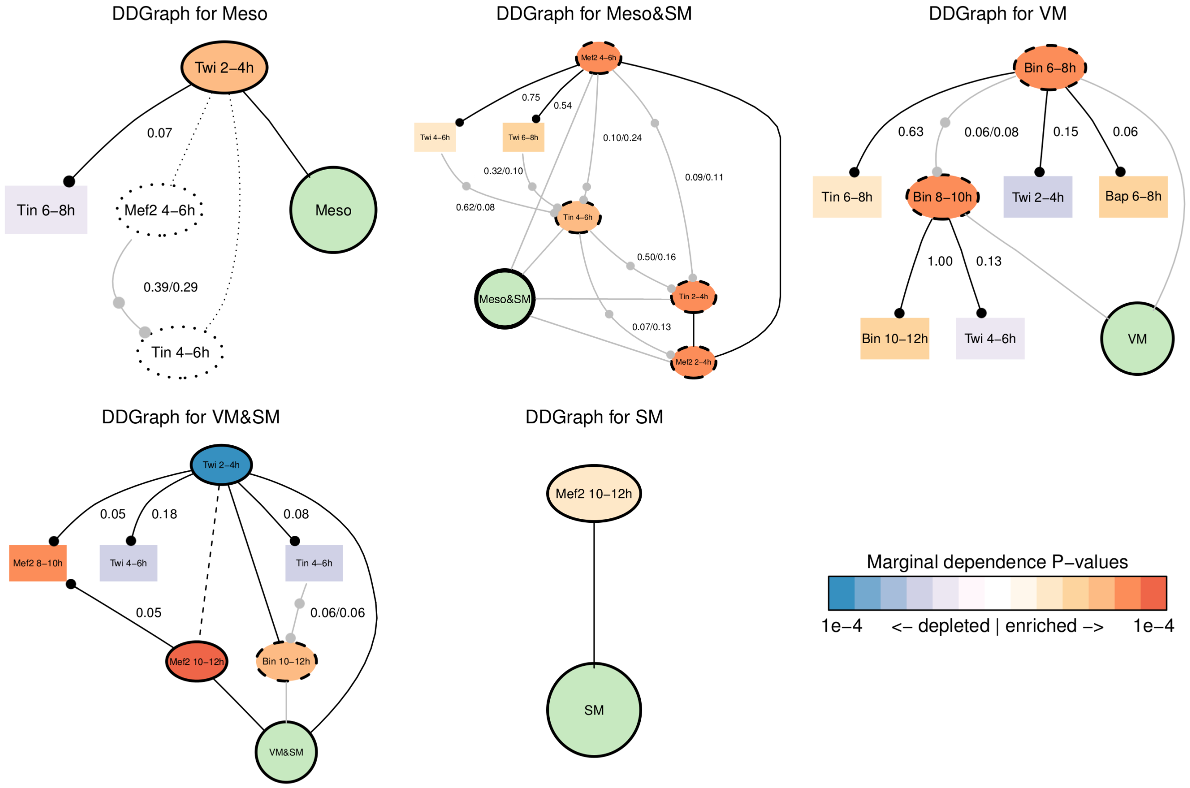

Supplementary Figure S7. DDGraph for results of NCPC* on 5 CRM classes of mesoderm at $\alpha = 0.05$. The result is the same as Figure 6 with the exception of Meso and VM&SM classes. For the Meso class we find two additional variables with conditional joint dependence. Following our results on synthetic data we discard them as false positives. For the VM&SM class we find that Bin 10-12h has not only joint dependence, but also conditional dependence (when conditioning on Twi 2-4h). This further confirms that Bin 10-12h is a member of the Markov blanket of VM&SM CRM class.

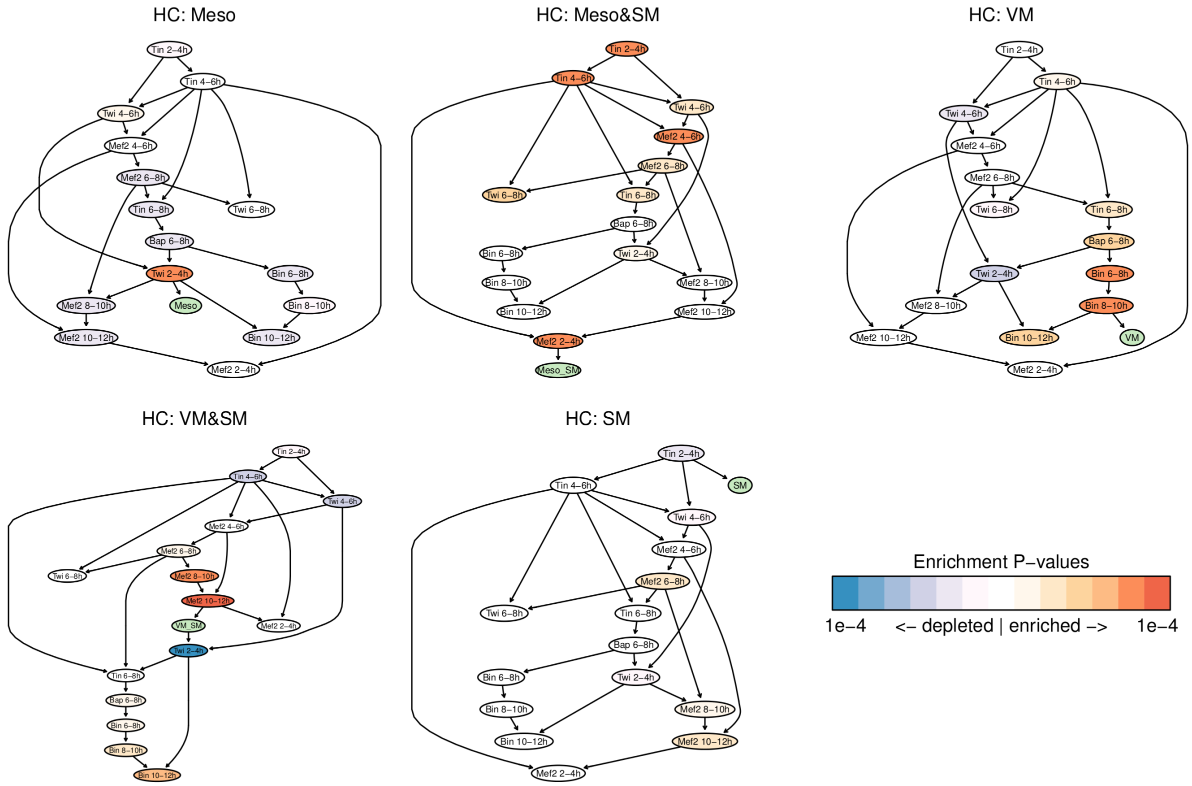

Supplementary Figure S8. Hill-climbing with BIC penalization applied to the 5 CRM classes. BIC penalization has a high false negative rate and it tends to finds subsets of variables we find with NCPC. However, in case of the SM class the variable picked to be the only causal parent does not make biological sense.

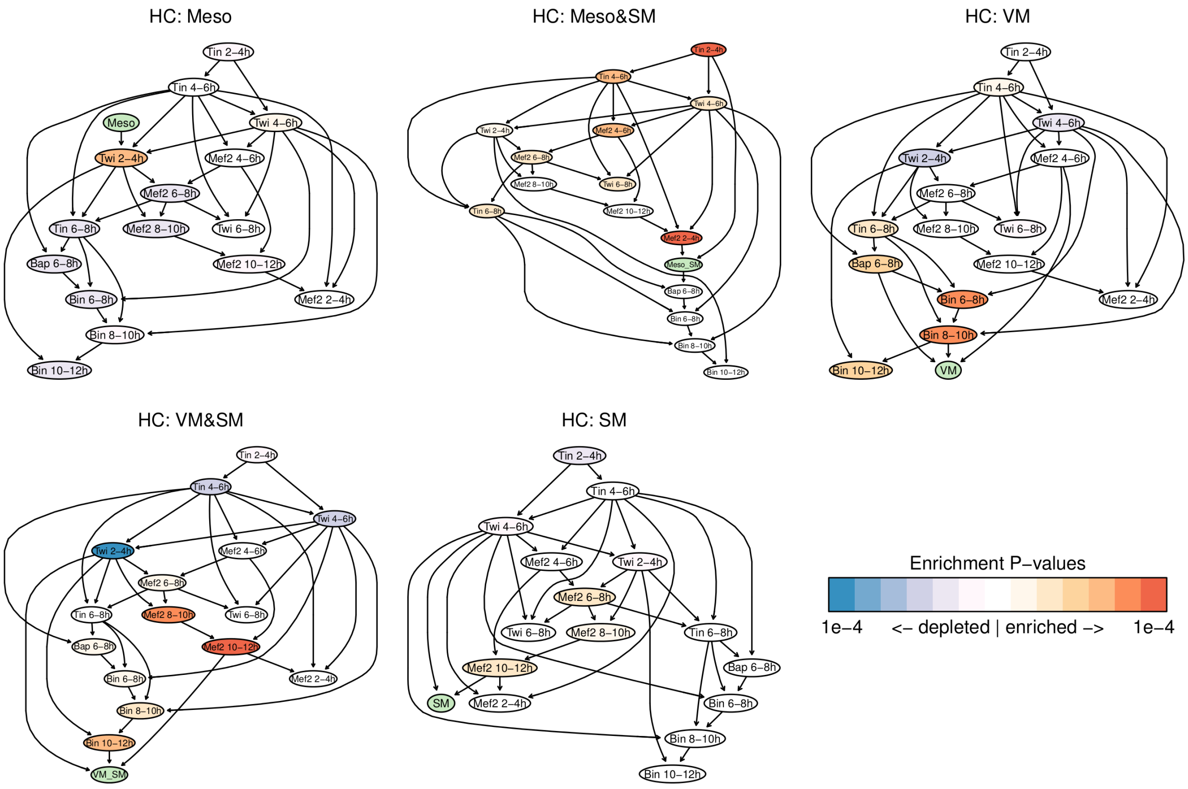

Supplementary Figure S9. Hill-climbing with BDe penalization applied to the 5 CRM classes. BDe penalization has a high false positive rate and it tends to finds supersets of variables we find with NCPC.

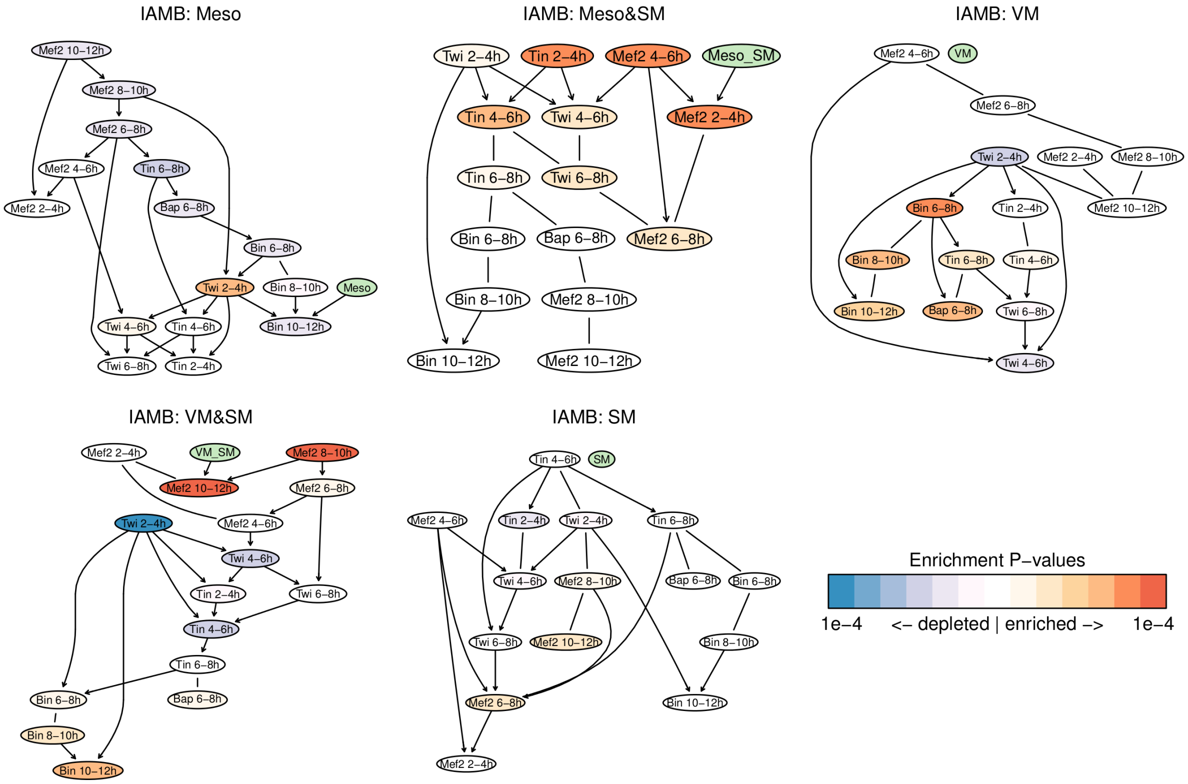

Supplementary Figure S10. IAMB applied to the 5 CRM classes at α = 0.05. IAMB finds similar, but sometimes seemingly wrong causal neighbours. For instance, for Meso it finds that Bin 10-12h has direct dependence (being causal child to Meso CRMs), while Twi 2-4h is a causal spouse, and thus independent of Meso CRMs. However, the situation is exactly reverse, Bin 10-12h being independent of Meso CRMs and Twi 2-4h having strong dependence on Meso CRMs. For VM and SM IAMB doesn't find any variables that directly influence these CRMs.

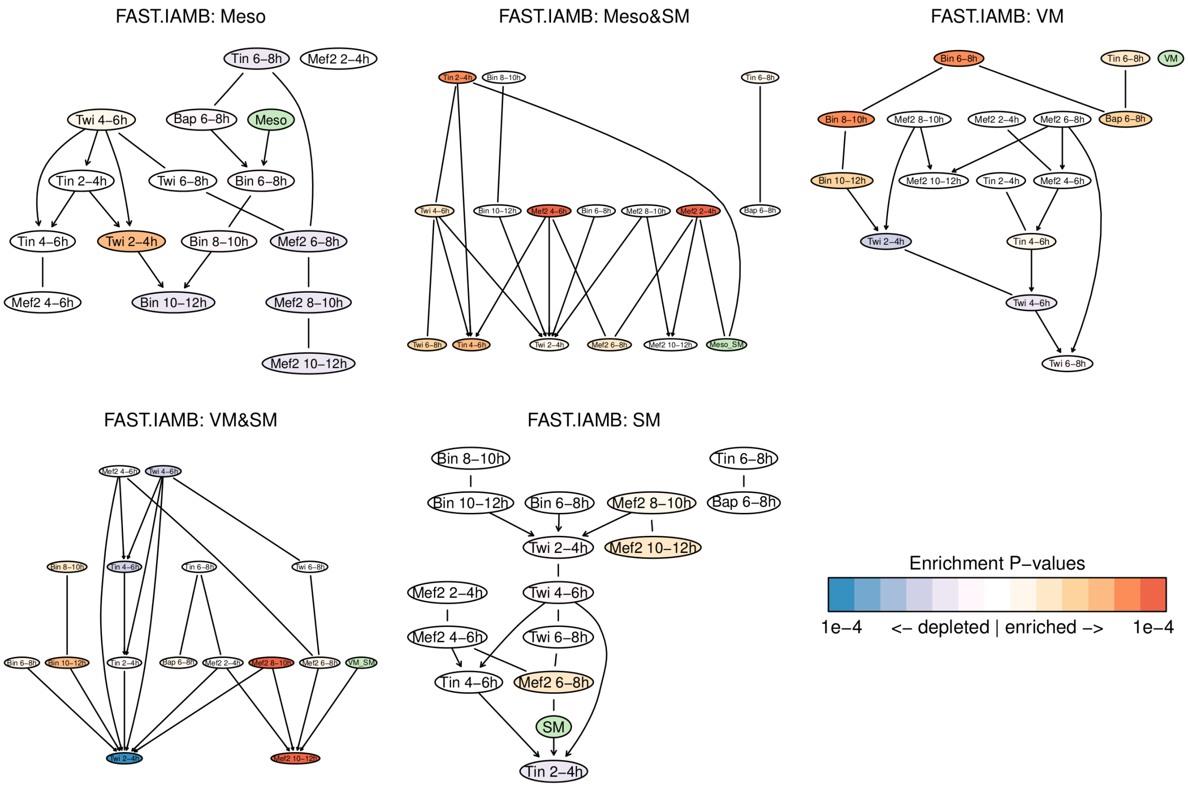

Supplementary Figure S11. FastIAMB applied to the 5 CRM classes at $\alpha = 0.05$. Fast IAMB compared to IAMB finds variables that further violate the dependencies found in the data.

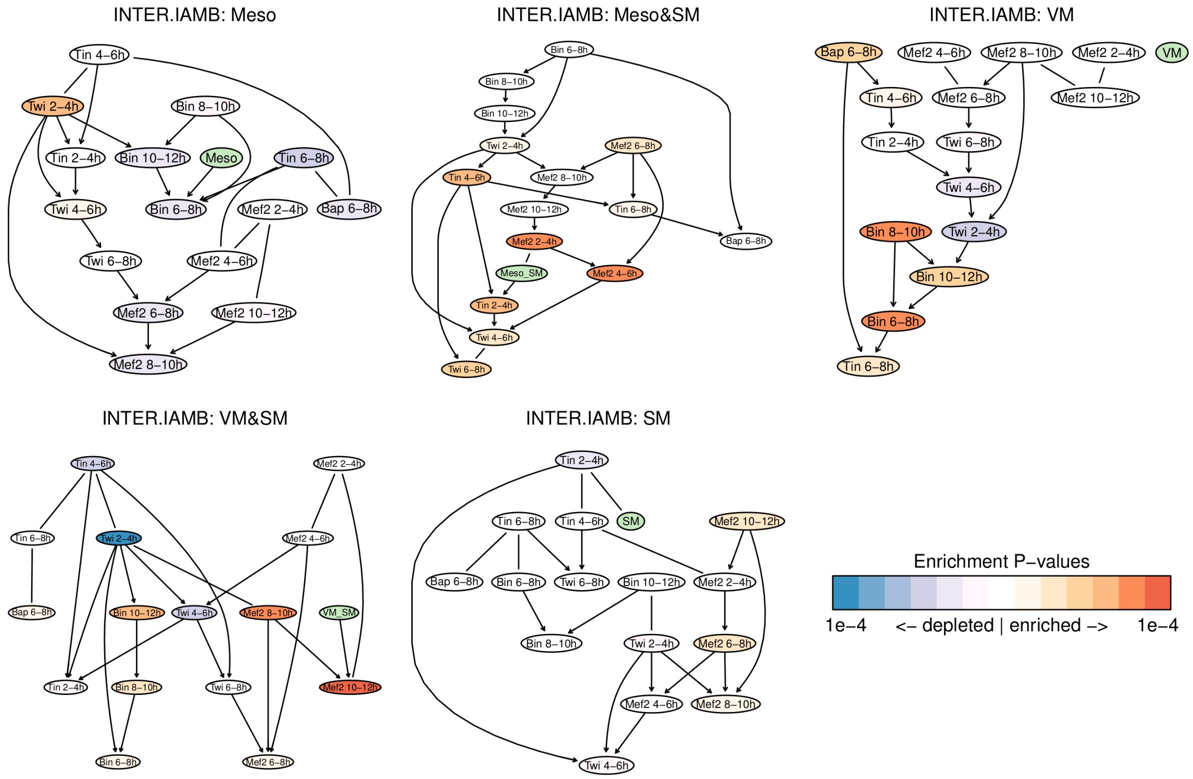

Supplementary Figure S12. InterIAMB applied to the 5 CRM classes at α = 0.05. InterIAMB finds variables similar to other IAMB class of algorithms. In case of Meso it also doesn't find Twi 2-4h as having direct dependence.

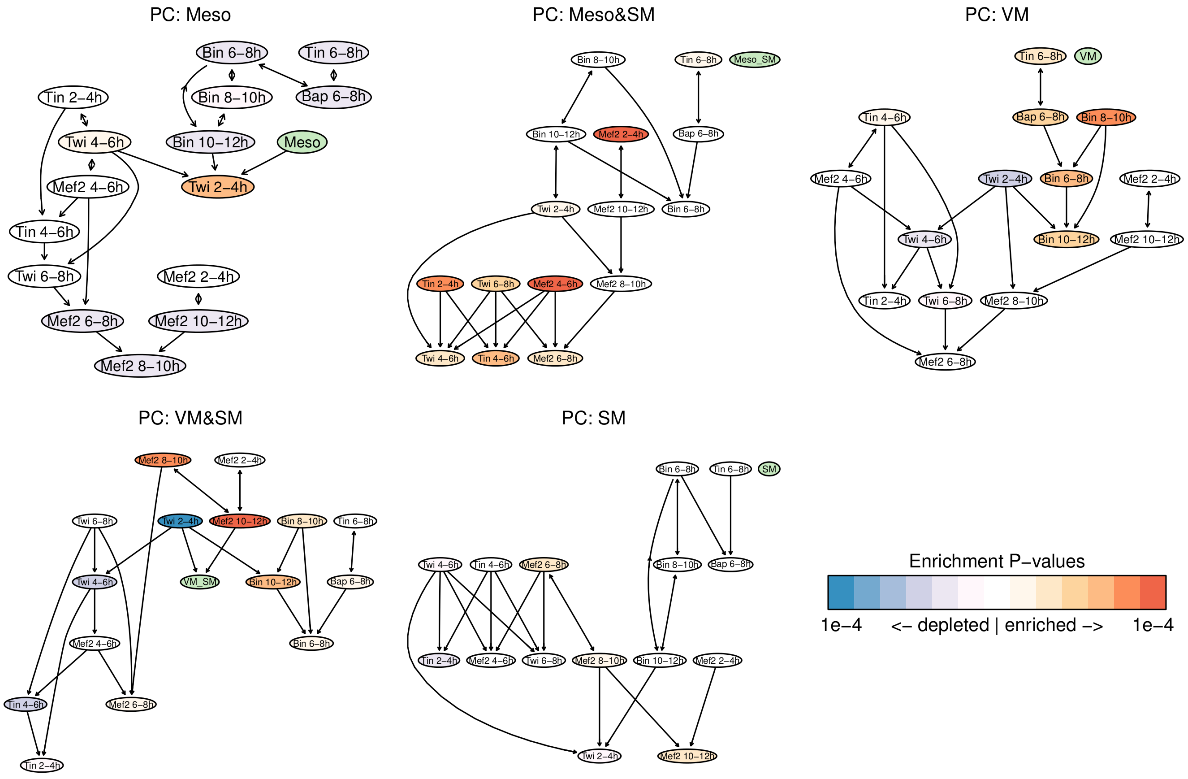

Supplementary Figure S13. PC algorithm applied to 5 CRM classes at α = 0.05. In 3 out of 5 cases the class labels node is disconnected to the graph, although it is clearly related to other features, as denoted by node colouring based on simple enrichment.

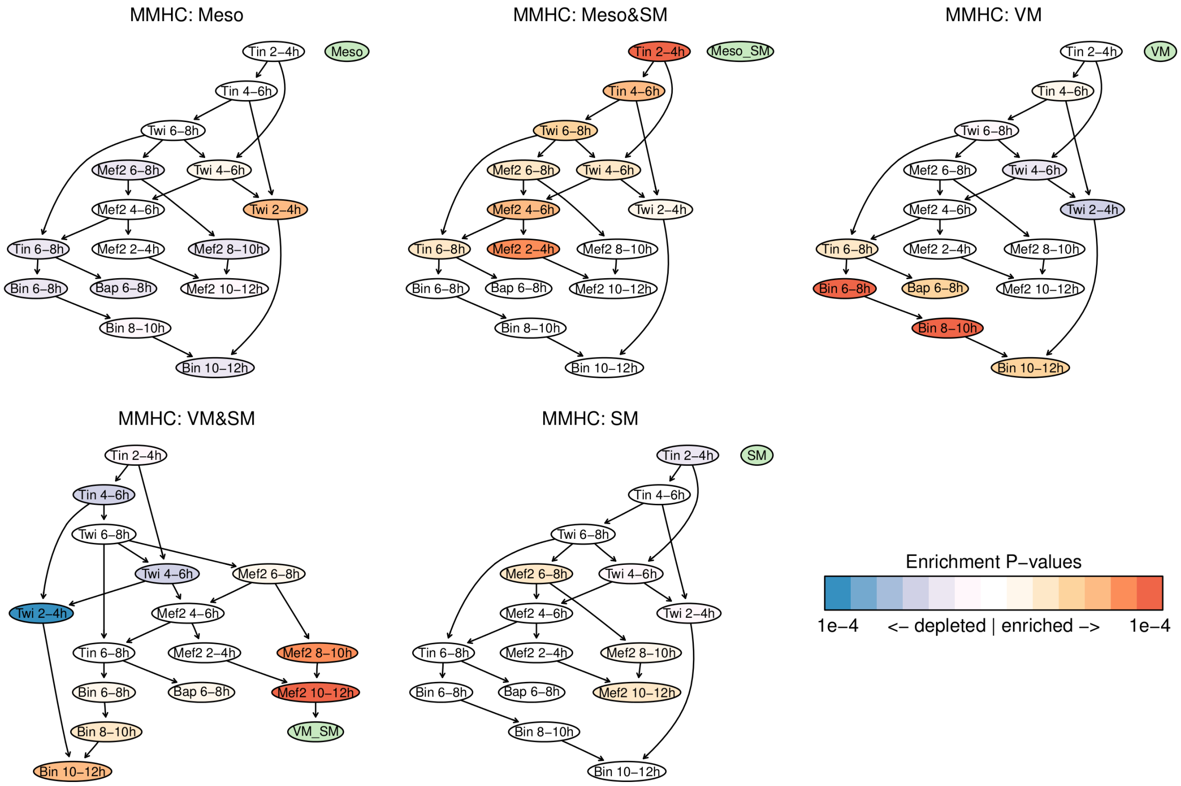

Supplementary Figure S14. MMHC with BIC penalization applied to the 5 CRM classes. Consistent with our results on synthetic data MMHC has the highest false negative rate, and in 4/5 cases the CRM class is disconnected in the graph.

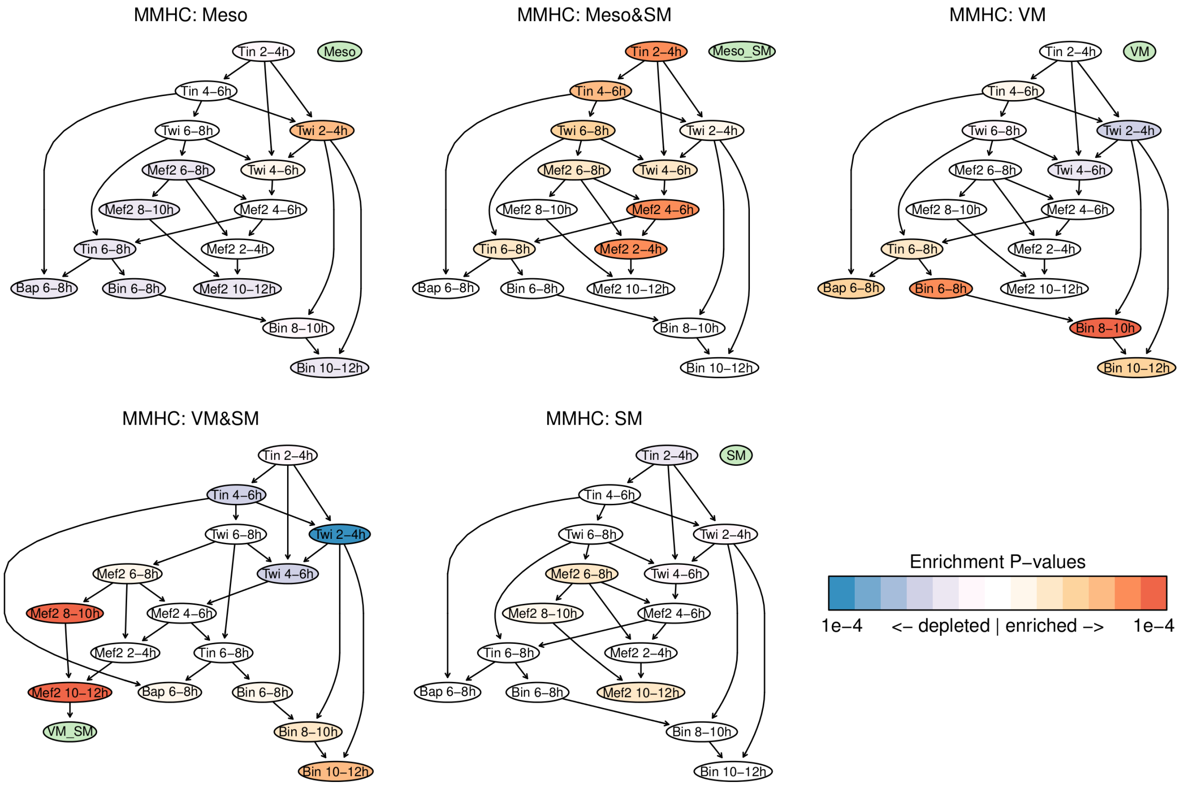

Supplementary Figure S15. MMHC with BDe penalization applied to the 5 CRM classes. Consistent with our results on synthetic data MMHC has the highest false negative rate, and in 4/5 cases the CRM class is disconnected in the graph.
